# Competitive docking model for prediction of the human nicotinic acetylcholine receptor α7 binding of tobacco constituents

**DOI:** 10.18632/oncotarget.24458

**Published:** 2018-02-08

**Authors:** Hui Wen Ng, Carmine Leggett, Sugunadevi Sakkiah, Bohu Pan, Hao Ye, Leihong Wu, Chandrabose Selvaraj, Weida Tong, Huixiao Hong

**Affiliations:** ^1^ Division of Bioinformatics and Biostatistics, Office of Research, National Center for Toxicological Research, U.S. Food and Drug Administration, Jefferson, AR 72079, USA; ^2^ Division of Non-clinical Science, Office of Science, Center for Tobacco Products, U.S. Food and Drug Administration, Silver Spring, MD 20993, USA

**Keywords:** prediction, tobacco constituents, addiction, molecular docking, nicotinic acetylcholine receptor

## Abstract

The detrimental health effects associated with tobacco use constitute a major public health concern. The addiction associated with nicotine found in tobacco products has led to difficulty in quitting among users. Nicotinic acetylcholine receptors (nAChRs) are the targets of nicotine and are responsible for addiction to tobacco products. However, it is unknown if the other >8000 tobacco constituents are addictive. Since it is time-consuming and costly to experimentally assess addictive potential of such larger number of chemicals, computationally predicting human nAChRs binding is important for in silico evaluation of addiction potential of tobacco constituents and needs structures of human nAChRs. Therefore, we constructed three-dimensional structures of the ligand binding domain of human nAChR α7 subtype and then developed a predictive model based on the constructed structures to predict human nAChR α7 binding activity of tobacco constituents. The predictive model correctly predicted 11 out of 12 test compounds to be binders of nAChR α7. The model is a useful tool for high-throughput screening of potential addictive tobacco constituents. These results could inform regulatory science research by providing a new validated predictive tool using cutting-edge computational methodology to high-throughput screen tobacco additives and constituents for their binding interaction with the human α7 nicotinic receptor. The tool represents a prediction model capable of screening thousands of chemicals found in tobacco products for addiction potential, which improves the understanding of the potential effects of additives.

## INTRODUCTION

Continuing to smoke increases the risk of diseases that are caused by the toxicants in tobacco smoke to smokers themselves [1, 2]. Furthermore, exposure to tobacco smoke in the environment leads to adverse health effects such as respiratory symptoms, impaired lung function, coronary heart disease, nasal irritation, stroke and lung cancer in nonsmokers as is seen in smokers [3–6]. The Third National Health Survey showed that a large fraction of the US population has detectable serum cotinine levels due to environmental tobacco smoke exposure [7]. The Surgeon General's Report and recent studies indicate that children exposed to tobacco smoke in the environment have a high likelihood of ear disease, respiratory symptoms, lower respiratory illness, and sudden infant death syndrome [8]. A longitudinal study of a large cohort of middle-aged and older adults found that many individuals, especially women, were passively exposed to tobacco smoke at home, public transport stations, workplaces, and recreational places [9]. Thus, tobacco smoke has detrimental effects for nonsmokers as well as smokers and is a public health issue.

Nicotinic acetylcholine receptors (nAChRs) belong to the superfamily of ligand-gated ion channels. Pentameric in nature, nAChRs consist of an extracellular domain approximately 200 amino acids in length (where the ligand binding domain [LBD] resides), a transmembrane region (consisting of four transmembrane helices), and an intracellular domain that connects the third and fourth transmembrane helices [10]. There are two major types of nAChRs: the neuronal and the muscle-type nAChRs; the former have been a major target in studies of addiction arising from tobacco use. The neuronal nAChRs formed by 12 known subunits, α2-α10 (n.b. α8 found in avian family and not mammals) and β2-β4 [10, 11]. The α7-α10 subunits can form functional homopentamers, while the α2-α6 require other α or β subunits to form a functional heteropentamer [11–14]. The α7 nAChR has been found to play a role in reinforcing and inducing nicotine dependence [15–19].

Various studies have identified thousands of chemicals in tobacco smoke, with the highest estimate at over 9,500 chemicals [20]. While the harmful effects of some tobacco constituents, such as the list of 93 harmful and potentially harmful constituents (HPHCs) established by the FDA, have been well studied, the biological effects of the majority of tobacco constituents remain unknown. Accordingly, understanding the effects of these chemicals on the body may be useful for strategies to reduce tobacco product harm.

The biological effects of tobacco constituents are typically tested with a battery of laboratory experiments. However, acquiring experimental data on the biological actions of these chemicals is both time-consuming and costly. *In silico* techniques offer a rapid approach to study and prioritize laboratory experiments needed to study tobacco constituents. Among available computational methods such as pharmacophore modeling [21–24], comparative molecular field analysis [25], decision tree [26], decision forest [27–33], support vector machine [34, 35], and other machine learning methods [36–38], molecular docking is one of the most established and widely-used approaches to assess the binding activity of chemicals. Molecular docking involves the prediction of how chemicals interact with proteins [39–42]. Knowing the binding potential of a chemical is important as receptor binding often initiates a cascade of chemical-induced biological actions. Apart from predicting how a chemical binds to the active site of a receptor, the inherent flexibility of proteins in ligand-receptor recognition is another important factor that needs to be considered as the conformation of a protein is closely linked to its function. Many times, upon binding to a ligand, a protein changes its conformation to perform different functions in complex biological processes [43]. However, majority of docking studies are still performed under the “fully flexible chemical vs. rigid protein” condition due to the high computational cost required to allow modeling flexibility. Indeed, even limited flexibility introduced to the protein (i.e., only on a few key residues in the active site) considerably increases the calculation time in the docking of a given chemical. Therefore, assessing a large library of compounds with molecular docking using fully flexible proteins is impractical.

Our previous study investigated the interactions of chemicals with the ligand binding domain of the α4β2 nAChR [44]. A similar study was conducted for the LBD of the α7 nAChR (α7 nAChR-LBD), not only to investigate how chemicals interact with the receptor but also to develop a model to predict the potential binding of chemicals to α7 nAChR-LBD. To date, the three dimensional (3D) structure of human α7 nAChR-LBD has not been elucidated experimentally. The closest available 3D structure to human α7 nAChR-LBD is α7 nAChR chimera (Protein Data Bank (PDB) ID: 3SQ6). Therefore, it would be useful to construct a 3D structure of human α7 nAChR-LBD, but also for a prediction model that incorporates the protein flexibility involved in the ligand-receptor recognition process. Here, we describe the development of a competitive docking model (CDM) based on an approach similar with our previously published model [40] for predicting estrogen receptor binding activity [32, 45] to help mitigate the shortcoming of rigid-protein docking by favoring the more energetically favorable receptor-ligand complex. The ability of CDM to predict the binding of chemicals to human α7 nAChR-LBD was assessed with a set of compounds whose human α7 nAChR-LBD binding had been experimentally tested. Finally, the important interactions that occurred when the receptor was bound by different chemicals were also investigated with molecular dynamics (MD) simulations [32, 46–48]. We elucidated the potential key residues in human α7 nAChR-LBD that take part in chemical binding, and revealed good performance of the model in chemical binding prediction. This model focuses on the interaction of these molecules with the receptor's ligand binding domain, but does not account for interactions at other receptor sites that may modulate receptor activity. This α7 nAChR binding activity prediction model may be useful for screening tobacco constituents that may have addiction potential as well as for regulatory priority setting for laboratory testing these compounds.

**Figure 1:**
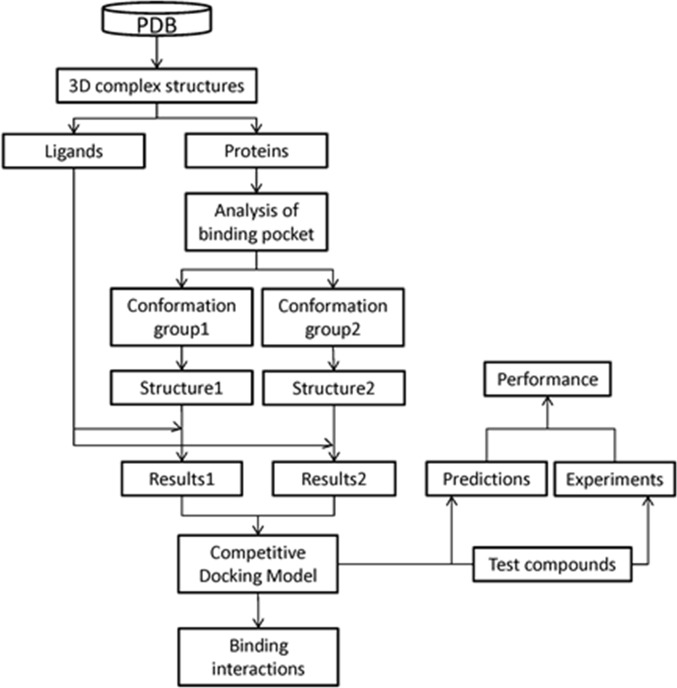
Overall modeling scheme The 3D structures of complexes of nAChBPs with different ligands were retrieved from the PDB. Ligands and proteins were separated. Binding pocket similarity was analyzed, resulting two groups. One template structure was selected from each group based on sequence similarity with human nAChR. Two 3D structures of the human α7 nAChR-LBD were constructed using homology modeling based on the selected templates. Using these two structures, the CDM was developed with the training set ligands. The CDM was used to predict the binding activities of the test set compounds. The prediction results were validated through experimental determination of the binding activities of the test set ligands.

Figure 1 gives the overview of this study. The CDM, which accounts for protein flexibility, was developed to predict the binding potential of chemicals to the human α7 nAChR-LBD. Developed using a training set of ligands obtained from the PDB, the CDM was used to predict, with good accuracy, the binding potential for a set of test compounds whose binding activity was experimentally validated. The potential key ligand binding interactions of human α7 nAChR were elucidated using MD simulations. These interactions include non-polar interactions (e.g., hydrophobic contacts, pi-pi interactions), as well as polar interactions (e.g., hydrogen bonding, pi-cation interactions). Key residues involved in these binding interactions have been identified. The elucidated binding interactions clarified the understanding of chemical binding potential to human α7 nAChR. The developed CDM shows applicability in computational high-throughput screening of tobacco constituents with addiction potential and may play a role in efforts to protect and improve public health.

## RESULTS

### Analysis of ligand binding pockets and selection of templates for homology modeling

**Figure 2:**
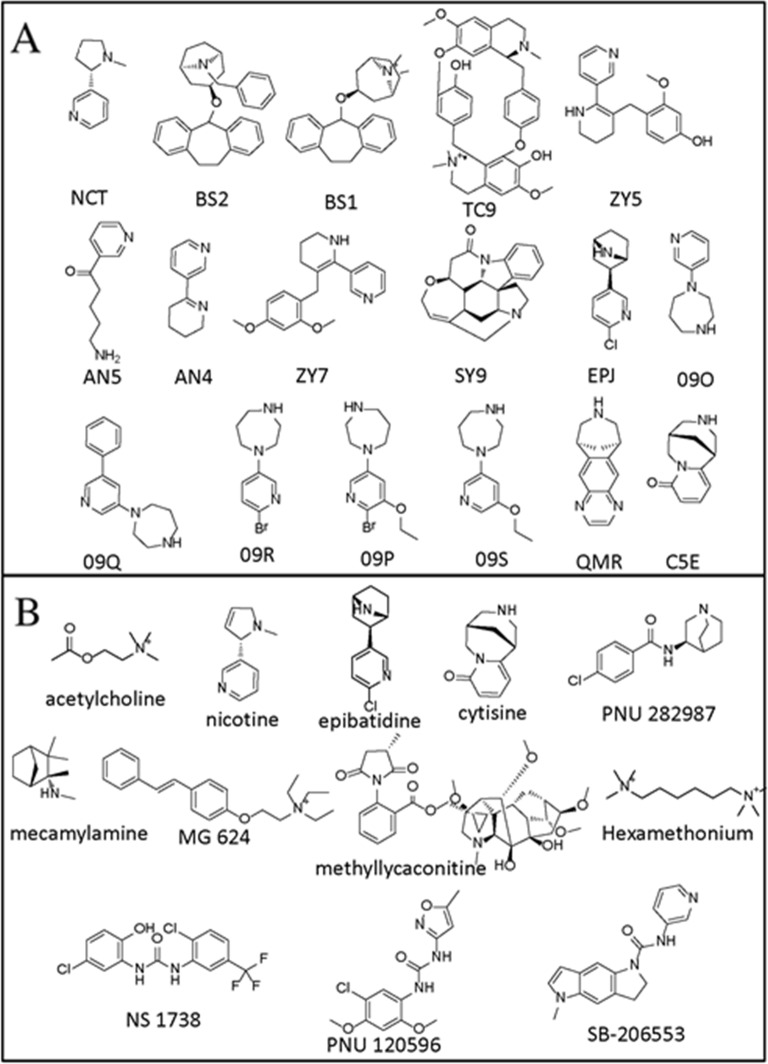
Structures of the compounds The training **(A)** and test **(B)** compounds identifications that were used in this paper are noted below the structures.

The PDB complexes (PDB IDs and the associated ligands) that were chosen as potential templates for constructing human α7 nAChR-LBD are listed in Table 1. The receptors in these PDB structures were in complex with ligands (Figure 2) that were found to have human α7 nAChR binding data (with the exception of AN4 and AN5 bound to 2WNL, which had rat α7 nAChR binding data). Among these, 16 complexes were acetylcholine binding proteins (AChBP), while the remaining complex was a chimeric nAChR-LBD. Apart from the latter, which shared the highest percent sequence identity (63%) with human nAChR-LBD, all other templates were found to share approximately 24-26% sequence identity with the target. While three of these complexes were resolved at a resolution below 2 Å (2WNJ, 2WN9, 2XYS), the majority of the structures had a resolution of 2 to 3 Å.

**Table 1:** The PDB complexes shortlisted as potential templates to construct the human α7 nAChR-LBD, with associated details and available experimental α7 nAChR binding data

PDB ID	Ligand ID	Protein	Species	α7 binding data^*^		Sequence identity (%)	Resolution (Å)
				Ki(nM)	Ref.		
3SQ6	EPJ	α7-nrc^*^	HS, LS	18	47	63.24	2.80
1UW6	NCT	AChBP	LS	170±65	47	24.75	2.20
3U8J	09O	AChBP	LS	136	47	23.88	2.35
3U8L	09Q	AChBP	LS	>10000	47	23.88	2.32
3U8M	09R	AChBP	LS	190	47	23.88	2.70
3U8K	09P	AChBP	LS	9970	47	23.88	2.47
3U8N	09S	AChBP	LS	847	47	23.88	2.35
2W8G	BS2	AChBP	AC	79.4	48	26.24	2.60
2W8F	BS1	AChBP	AC	79.4	48	26.24	2.70
2XYT	TC9	AChBP	AC	2975	49	26.24	2.05
2WN9	ZY5	AChBP	AC	235^&^	50	25.24	1.75
2XYS	SY9	AChBP	AC	4854	49	26.24	1.91
2WNL	AN5	AChBP	AC	200^&^	50	25.24	2.70
2WNL	AN4	AChBP	AC	200^&^	50	25.24	2.70
2WNJ	ZY7	AChBP	AC	130	50	25.24	1.80
4AFT	QMR	AChBP	AC	322	51	26.24	3.2
4BQT	C5E	AChBP	AC	4200	51	26.24	2.88

Legends: ^*^ α7 nAChR binding data from human except AN4 and AN5 from rat; & Kd (nM).

Abbreviations: α7-nrc: α7-nicotinic receptor chimera; AChBP: Acetylcholine Binding Protein; HS: Homo sapiens; LS: Lymnaea stagnalis; AC: Aplysia Californica.

**Figure 3:**
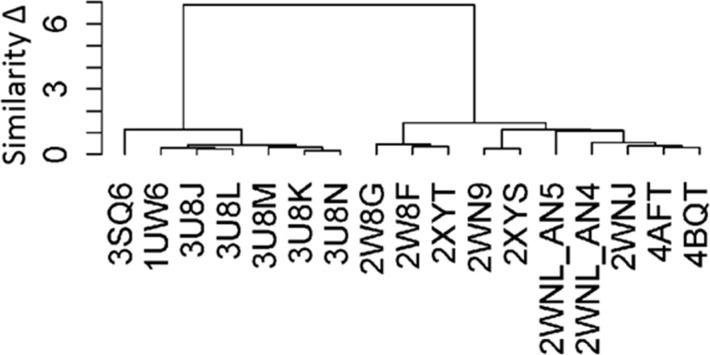
The hierarchical clustering of the binding pockets of 17 complex structures The similarity (y-axis) was measured using the RMSDs between the pocket conformations. The PDB ID (x-axis) was used to indicate the complex.

Figure 3 depicts the results of the clustering analysis performed on the ligand binding pockets of the 17 PDB complexes. These complexes were clustered based on the RMSD of the residues lining their ligand binding pockets. Two distinct groups of protein conformations were observed; one representative complex was selected from each of the two groups (3SQ6 and 2XYT) as templates for construction of human α7 nAChR-LBD structures. The complex structure 3SQ6 had the highest sequence identity with human α7 nAChR-LBD and thus was selected as a template for the homology modeling; the complex structure 2XYT was selected as another template for the homology modeling mainly due to its good resolution (2 Å) combined with the relatively rigid and large ligand (TC9). The main difference between the two complexes was that the receptor conformation of the complex 3SQ6 had a more enclosed/smaller ligand binding pocket compared to the receptor conformation of the complex 2XYT, which had a more open/larger ligand binding pocket.

### 3D human α7 nAChR-LBD structures

**Figure 4:**
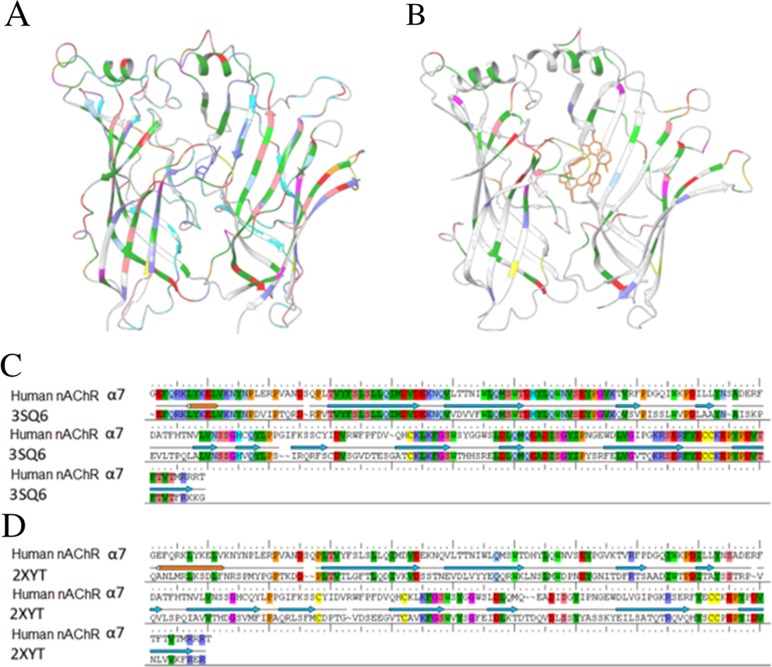
The template structures used in homology modeling of human α7 nAChR-LBD structures 3D structures are shown for 3SQ6 **(A)** and 2XYT **(B)**. The alignment results of the sequence of the human α7 nAChR with the template proteins in 3SQ6 **(C)** and 2XYT **(D)** are shown. The colored regions in a-d indicate the regions that were well-aligned between the human α7 nAChR and 3SQ6 or 2XYT.

Figure 4 shows the structures 3SQ6 and 2XYT and alignments of their protein sequences with the primary sequence of human α7 nAChR. The colored sections indicate the regions containing identical residues between the templates and human α7 nAChR. The protein in 3SQ6 shared a high sequence identity (63%) with human α7 nAChR-LBD (Figure 4A and 4C), while the protein in 2XYT shared 26% sequence identity with human α7 nAChR-LBD (Figure 4B and 4D). It was noted that the loop sections and the secondary structure regions (α-helices and β-sheets) of the protein in 3SQ6 aligned better with human α7 nAChR-LBD than those of the protein in 2XYT.

**Figure 5:**
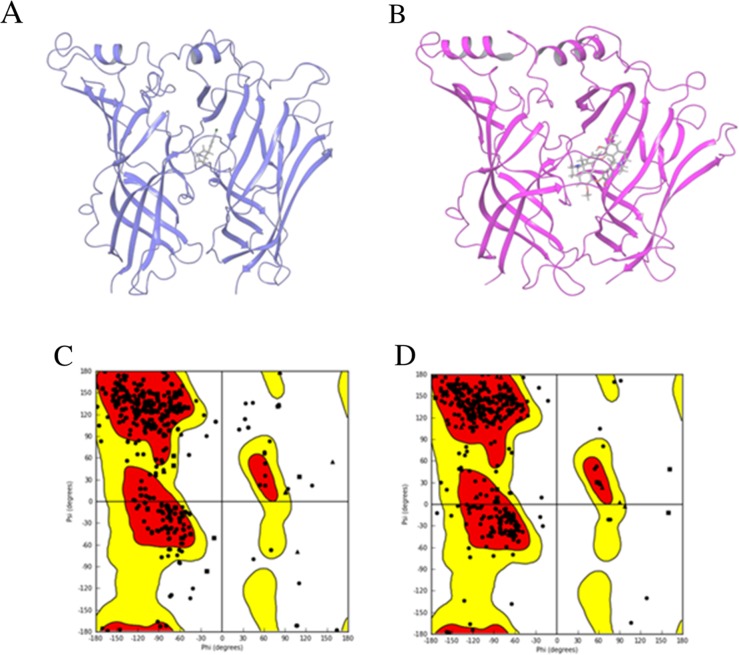
Homology modeling results The initial structures of the human α7 nAChR-LBD constructed using homology modeling based on the template structures 3SQ6 **(A)** and 2XYT **(B)**. The proteins were drawn in ribbon models and the ligands were plotted in stick models. The Ramachandran plots are given for the corresponding initial structures based on 3SQ6 **(C)** and 2XYT **(D)**. The x-axis and y-axis indicate the two dihedral angles of amino acid residues in the homology model structures. Each point represents an amino acid residue. The favored regions are color-coded in red, the allowed regions in yellow, and the not allowed regions in white.

Figure 5 displays the initial 3D human α7 nAChR-LBD structures constructed by homology modeling using 3SQ6 (Figure 5A) and 2XYT (Figure 5B) as the templates. The Ramachandran plot of the initial 3D structure constructed from 3SQ6 (Figure 5C) shows that 92.2% of residues were placed in the favored zone (the red regions) and 99.1% of residues were placed in the allowed zone (the red and yellow regions), indicating that the 3D structure is of good quality and at a stable state. In the same way, the Ramachandran plot depicted in Figure 5D shows that 92.4% of residues were placed in the favored zone (the red regions) and 99.2% of residues were placed in the allowed zone (the red and yellow regions), indicating the initial homology structure based on 2XYT is of good quality and at a stable state.

**Figure 6:**
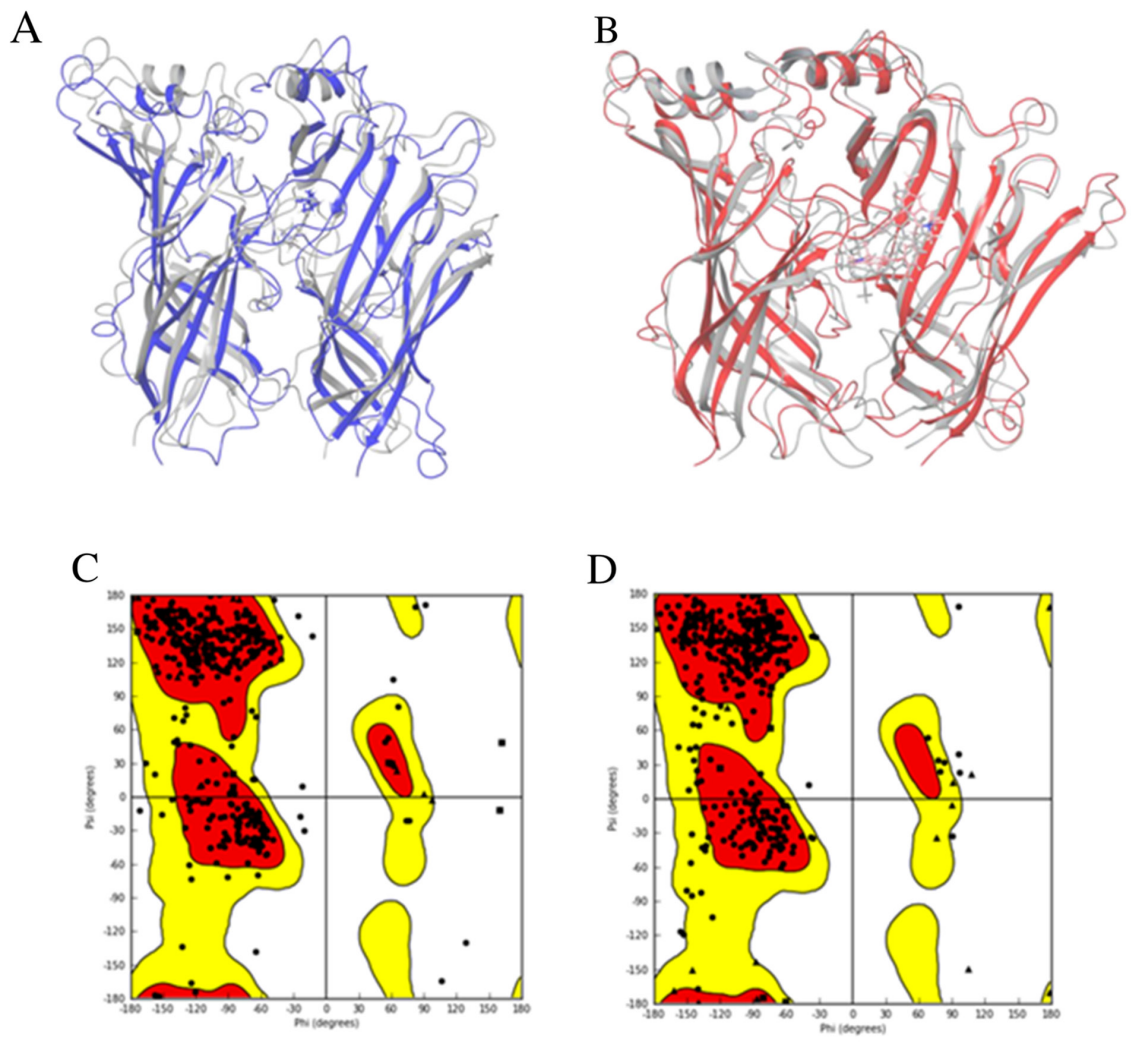
Optimized structures The optimized structures of the human α7 nAChR-LBD based on the template structures 3SQ6 **(A, purple)** and 2XYT **(B, red)**. The initial homology structures are shown in grey. The Ramachandran plots are given for the corresponding optimized structures based on 3SQ6 **(C)** and 2XYT **(D)**. The favored regions are color-coded in red, the allowed regions in yellow, and the not allowed regions in white.

The initial 3D structures were optimized through MD simulations. The optimized 3D structures of human α7 nAChR-LBD constructed from 3SQ6 and 2XYT were superimposed with the initial 3D structures from the homology modeling as shown in Figure 6A and 6B, respectively. Comparison of the optimized structures with their initial structures indicates that the MD simulations led to relatively larger changes in the conformation of the loops, while the helixes had very small changes in the optimizations. The Ramachandran plot of the optimized 3D structure from 3SQ6 (Figure 6C) shows that 93.1% and 99.4% of residues were placed in the favored zone (the red regions) and the allowed zone (the red and yellow regions), respectively, higher than the percentages of residues (92.2% in the favored zone and 99.1% in the allowed zone) for the initial structure, indicating the optimized 3D structure is of a higher quality and at a more stable state. The Ramachandran plot of the optimized 3D structure from 2XYT (Figure 6D) shows that 93.7% and 99.6% of residues were placed in the favored zone (the red regions) and the allowed zone (the red and yellow regions), respectively, higher than the percentages of residues (92.4% in the favored zone and 99.4% in the allowed zone) for the initial structure, revealing the optimized 3D structure is of a higher quality and at a more stable state.

### Competitive docking model

The results of docking the 17 ligands in the training set to the two human α7 nAChR-LBD structures are presented in Table 2. Three ligands (ID: TC9, BS1, BS2) were only docked in the human α7 nAChR-LBD structure constructed from 2XYT, and their docking scores were used in the development of the CDM. The other 14 ligands were successfully docked to both human α7 nAChR-LBD structures. According to the CDM rules, the lower docking scores were used for training the CDM. It was noted that the lower of the two docking scores for most of the ligands came from docking to the 3SQ6-based human α7 nAChR-LBD, except for SY9, which was the only ligand that obtained a lower docking score in the 2XYT-based human α7 nAChR-LBD structure than in the 3SQ6-based human α7 nAChR-LBD structure.

**Table 2:** Docking scores for the training set ligands obtained from docking to both 3SQ6-based and 2XYT-based α7 nAChR-LBD structures as well as the winning score

ID	XPGscore^*^		Winning score
	3SQ6-based α7 nAChR-LBD	2XYT-based α7 nAChR-LBD	
EPJ	−8.725	−5.2	−8.725
ZY5	−8.082	−5.601	−8.082
ZY7	−8.047	−5.537	−8.047
09S	−8.016	−4.093	−8.016
09Q	−7.704	−5.072	−7.704
AN5	−7.673	−4.681	−7.673
09R	−7.666	−5.109	−7.666
NCT	−7.512	−3.1	−7.512
09O	−7.439	−3.656	−7.439
QMR	−7.353	−4.135	−7.353
09P	−7.257	−3.616	−7.257
AN4	−6.982	−4.674	−6.982
C5E	−6.362	−3.65	−6.362
TC9	ND	−5.583	−5.583
BS1	ND	−5.015	−5.015
BS2	ND	−4.36	−4.36
SY9	−3.596	−4.218	−4.218

Abbreviations: ^*^ XPGscore: Extra-precision Glide docking score.

With the 17 winning docking scores listed in the last column of Table 2 as the training data, the predictive CDM was constructed using and is expressed by equation (1).

$$\text{CDM}=1-\frac{\text{score}+8.725}{4.507}$$ (1)

### Key ligand binding interactions

The 17 human α7 nAChR-LBD-ligand complexes from the CDM were subjected to 20 ns MD simulations to investigate potential key ligand binding interactions. The RMSD plots of the MD simulations are shown in Supplementary Figure 1. These plots indicate that the 17 human α7 nAChR-LBD-ligand complexes reached a more or less stable state throughout the 20 ns MD simulations, following the sharp increase in the initial stage.

The key binding interactions and the residue fluctuations of human α7 nAChR-LBD were investigated using the frames from the last ns of the MD simulations. Supplementary Figure 2 shows the RMSF values that depict the fluctuations of residues of human α7 nAChR in the MD simulations. In general, the fluctuations for the residues remained between 0.5-2Å. Larger fluctuations were observed in the loop regions compared to the α-helices and β-sheets, as expected; human α7 nAChR complexes bound with ligands AN4, AN5, BS2 and 09R showed slightly more restricted movement compared to the complexes bound with other ligands, which may be due to the small size and rigid structures.

The key binding interaction fractions calculated over the last 1 ns are shown in Supplementary Figures 3 for hydrophobic contact, S4 for pi-pi interactions, and 5 for hydrogen bonds and pi-cation (See Supplementary Figures 6–22 for the diagrams of interactions for the individual ligands). Table 3 summarizes the important residues involved in the ligand binding of human α7 nAChR. The residues involved in hydrophobic contacts were Tyr93, Phe104, Val108, Leu109, Tyr118, Leu119, Pro121, Trp149, Tyr188 and Tyr 195 in chain A, and Trp55, Tyr93, Leu109, Leu119, Trp149, Ile169, Pro170, Tyr188, Cys190 and Tyr195 in chain B. Among these residues, the most important residue was Trp149 of chain B, which was involved in hydrophobic contact for most of the ligands. The other hydrophobic interaction residues were Tyr195 of chain B and Leu119 of chains A and B. The residues involved in pi-pi interactions were Phe104, Trp149, Tyr195 and Tyr188 in chain A and Trp55, His115, Trp149, Tyr195 and Tyr188 in chain B. The residues involved in hydrogen bonding interactions were Arg79, Asn107, and Leu119 in chain A and Arg79, Gln117, Trp149, Asp164, Ser166, Tyr188, and Tyr195 in chain B. Hydrogen bonding with the backbone of Leu119 of chain A was particularly important – eight of the 17 ligands formed hydrogen bonds with this residue. Interestingly, hydrogen bonding interactions were not found for BS1, BS2, EPJ, and QMR. In addition to hydrogen bonding, another polar interaction type (i.e., pi-cation) was observed between some ligands (BS1, ZY5, ZY7 and AN4) and human α7 nAChR-LBD. While the pi-cation interaction between human α7 nAChR-LBD and BS1 arose from the interactions between the positive charge found in the quaternary nitrogen of the ligand and the two tyrosine residues (Tyr188 and Tyr195) in chain A of the receptor, the pi-cation interactions between human α7 nAChR-LBD and ZY5, ZY7 and AN4 arose from the interactions between the aromatic rings in these residues with the positive charge of Arg79.

**Table 3:** Potential key binding interactions obtained from MD simulations performed on the docked complexes of the training set ligands

Interactions	Protein	Residues
Hydrophobic contact	A Chain	Leu119
	B Chain	Leu119, Trp149, Tyr195
Pi-pi	B Chain	Trp149, Tyr188
H-bonding	A Chain	Leu119
Pi-cation	A Chain	Arg79, Tyr188, Tyr195

### CDM predictions of test compounds

Human α7 nAChR binding activity was predicted for the 12 test compounds with the CDM expressed by equation (1). MG624 and hexamethonium were docked only to the 2XYT-based human α7 nAChR-LBD and the docking scores were used in the CDM predictions. The remaining 10 compounds were successful in molecular docking to both structures and, thus, the lower docking scores were used in the CDM predictions as listed in Table 4. The CDM prediction value for hexamethonium was −0.318 and, thus, hexamethonium was predicted as a non-binder. Hexamethonium exhibited binding affinity for nicotine binding sites, but did not show inhibition in methyllycaconitine binding, demonstrating that hexamethonium interacts with α4β2 but not α7 receptor subtypes, respectively [49]. The CDM prediction values for the remaining 11 compounds were positive (see the last column of Table 4) and, thus, these compounds were predicted as binders of human α7 nAChR. Out of these 11 predicted α7 binders, acetylcholine [50], epibatidine [51], nicotine [52], PNU282987 [53], methyllycaconitine [54], NS1738 [55], PNU120596 [56], and SB206553 [57] were confirmed by the experimental data reported in the literature.

**Table 4:** Binding activity prediction results of the test set ligands. A positive CDM prediction score indicates that the ligand is a human α7 nAChR-LBD binder while a negative score indicates otherwise

Ligands	XPGScore		Winning score	CDM
	2XYT-based α7 nAChR-LBD	3SQ6-based α7 nAChR-LBD		
Acetylcholine	−4.68	−4.812	−4.812	0.132
epibatidine	−5.2	−8.725	−8.725	1.000
nicotine	−3.1	−7.512	−7.512	0.731
cytisine	−3.65	−6.362	−6.362	0.476
PNU282987	−4.518	−5.878	−5.878	0.368
mecamylamine	−3.564	−4.873	−4.873	0.145
MG624	−4.954	failed to dock	−4.954	0.163
methyllycaconitine	−5.829	failed to dock	−5.829	0.357
Hexamethonium	−2.786	−1.001	−2.786	−0.318
NS1738	−7.108	−8.513	−8.513	0.953
PNU120596	−4.992	−6.188	−6.188	0.437
SB206553	−4.373	−6.671	−6.671	0.544

## DISCUSSION

Computational works such as MD simulations on nAChR α7 receptor of nonhuman species were reported [58, 59]. To date, the structure of a human α7 nAChR-LBD has yet to be elucidated. In order to inform the assessment of the addiction potential of tobacco constituents, 3D structures of human α7 nAChR-LBD are of great value, especially in the identification of tobacco constituents that have potential to bind human α7 nAChR. Knowledge of human α7 nAChR binding potential of tobacco constituents would be useful, as binding human α7 nAChR is a biological process that involves a cascade of biological responses in addiction and can be used for screening tobacco constituents with addiction potential. Therefore, the results could potentially be used to model a reduction in addiction potential of tobacco products by decreasing the amount of tobacco constituents that bind human α7 nAChR.

Homology modeling was used to generate the 3D structure of human α7. The template was selected based on the size of the ligand binding pocket of AChBP from different species. The 17 crystal structures were classified into two group. One template was selected from each group (3SQ6 and 2XYT) to model the human α7 nAChR. The size of the binding pocket of 2XYT was larger than 3SQ6 due to the binding of large ligand (TC9). The sequence alignments between the human α7 nAChR and the templates were performed using ClustalW from Maestro. The 3SN6 template had shown a higher sequence identity than 2XYT. Prime module from Maestro was used to build the 3D structure of human a7 nAChR. The generated models were assessed using Ramachandran plot. The 3SN6 and 2XYT based model structures had shown 99.1% and 99.2% residues in the allowed and favored regions in the Ramachandran plots. The initial models were subject to 100 ns molecular dynamics simulations to refine the side chain orientations and to relax the loop regions. The final frame from the trajectory file was selected as a representative structure. The refined models were better than the initial models in the Ramachandran plots and were used for subsequent molecular docking studies. Docking is a well-established method for assessing the binding potential of chemicals to the target receptor in the body. This technique has been used for predicting potential binders of nAChRs [60–64]. However, protein conformation flexibility remains an issue due to the rigid-body treatment of the protein structure during the rigid docking process, the most common docking protocol. A flexible docking protocol that allows changes in protein conformation is very expensive to implement. Application of flexible docking in a big number of molecules such as high-throughput screening is difficult, if not impossible, and currently is not a popular docking protocol due to the challenges of conformational sampling and energy weighting. Different methods have been developed to incorporate protein flexibility into molecular docking through balancing the computational cost and the conformational sampling space; for example, the flexible docking protocol uses experimentally-determined conformations to reduce conformation sampling space and to guide weighting energy of protein conformations. The CDM developed in this study was constructed in a similar way, taking into consideration the conformational flexibility inherent in proteins by incorporating the different protein (or more importantly, pocket) conformations most likely to be adopted. It is worth noting that issues related to potential false positive predictions are often found when using models based on the docking scores of CDM. These models utilize homology model structures based on docking scores used to measure the fitness of ligands in a binding pocket. Homology model structure are also determined by the orientation, shape, and energetic interactions of small molecules with the receptor with an approximation method for binding energy.

Clustering analysis of the templates shortlisted from the PDB was a key step in constructing 3D structures of human α7 nAChR in accounting for the protein flexibility in CDM. While structure 3SQ6 in PDB appeared to be the obvious template of choice for constructing human α7 nAChR-LBD, a second human α7 nAChR-LBD constructed based on structure 2XYT (despite the lower percentage sequence identity) demonstrated its usefulness during the docking of TC9, BS1 and BS2 (i.e., ligands that failed to dock to the 3SQ6-based human α7 nAChR-LBD but were known to bind to human α7 nAChR). This clearly showed the merit of using the CDM in the assessment of chemical binding potential to human α7 nAChR-LBD. More favorable scores were often observed in the 3SQ6-based rather than the 2XYT-based human α7 nAChR-LBD structures for majority of the ligands. This was not surprising, as the narrower binding pocket of the 3SQ6-based human α7 nAChR-LBD allowed the ligands to fit more snugly to the pocket (thus forming more interactions) compared to the 2XYT-based structure, which had a wider binding pocket opening.

Comparison of the residues in the active site of the human α7 nAChR structures that involved interactions with ligands revealed that 14 and 23 residues from Chain A and B interacted with the ligands. The interacting residues in Chain A and B are 148S, 149W, 150S, 151Y, 152G, 186R, 187F, 188Y, 190C, 191C, 192K, 193E, 194P, 195Y and 32Y, 33F, 34S, 55W, 56L, 57Q, 58M, 59S, 60W, 77T, 79R, 107W, 108V, 109L, 110V, 111N, 115H, 116C, 117Q, 118Y, 119L, 120P, 121P, respectively.

Binding interaction between nAChRs and some ligands were investigated experimentally [65, 72]. Apart from developing the CDM, this study also investigated potential key binding interactions between human α7 nAChR-LBD and the ligands in the training set. It was found that most ligands were anchored in the binding pocket of human α7 nAChR-LBD through rather non-specific hydrophobic contacts with a range of hydrophobic residues in the binding pocket. Residues Trp149 and Tyr195 in the B chain were found to interact strongly (i.e., observed through most of the frames extracted from the MD trajectories) with many of these ligands. Trp149 has been found to interact with ligands through hydrogen bonding and cation–π interactions in literature [66]. Leu119 in chain A was also found to interact with many of these ligands but with apparent weaker contacts. Considering that aromatic rings were contained in many residues of the binding pocket and most of the ligands, the formation of pi-pi interactions between human α7 nAChR-LBD and the ligands was expected. Experimental data confirmed binding interaction of Leu119 with large agonists of α7 [67]. Trp149 and Tyr188 in chain B were found to form rather stable pi-pi interactions with the ligands which were confirmed with the findings in the literature [68, 69, 71]. Similarly, polar interactions such as hydrogen bonding and pi-cation interactions were found to play an important role in receptor-ligand binding. In general, the hydrogen bonds observed were strong and were maintained in most of the trajectories. Similar with the hydrogen bonding interactions, the pi-cation interactions observed between the receptor (Arg79, Tyr188 and Tyr195 of chain A) and the ligands (BS1, ZY5, ZY7, and AN4) were also fair strong. While in the case of BS1, the positive charge originated from the ligand, the opposite was true for AN4, ZY5 and ZY7 (i.e., the aromatic rings from these ligands interacted with the positive charge from Arg79). With ZY5 and ZY7, the pi-cation interaction with Arg79 could be attributable to the electron-donating double hydroxyl/methoxy substituents of one of the aromatic rings. The mutation study demonstrated that Arg79 involved binding interaction with ligands of α7 [70].

## MATERIALS AND METHODS

### Overall modeling scheme

The overall modeling scheme of this study is depicted in Figure 1. First, the PDB was searched for suitable protein-ligand complexes to be used as potential templates to construct the 3D human α7 nAChR-LBD structures. During the search, only complexes containing ligands with known human α7 nAChR binding data were selected (see Table 1). These complexes were then subjected to a binding pocket analysis to examine the similarity or difference among the ligand binding pockets. Based on this analysis, two homology structures of human α7 nAChR-LBD were constructed. The CDM was developed using these 3D structures and the crystal-bound ligands (training set; see Figure 2A). The key binding interactions between the ligands and the 3D human α7 nAChR-LBD structures were examined with MD simulations. The model was then used to predict the binding activity of a series of test compounds (see Figure 2B) and the model prediction results were compared with the experimentally-obtained findings to assess model performance.

### Binding pocket analysis

Seventeen receptor-ligand complexes were obtained through the PDB search. The residues lining these ligand binding pockets were identified. Taking the PDB crystal structure 1UW6 as an example, the residues of its ligand binding pocket are: Asp86, Ala88, Tyr90, Lys140, Gly142, Ser143, Trp144, Thr145, His146, His147, Val184, Thr185, Tyr186, Ser187, Cys188, Cys189, Glu191, Ala192, Tyr193, Glu194, Lys241, Ile243, Trp260, Gln261, Gln262, Thr263, Gln280, Ser282, Thr306, Pro307, Leu309, Ala310, Arg311, Val312, Val318, Leu319, Tyr320, Met321, Pro322, Ser323, and Tyr371. Based on these residues, 17 3D structures of the α7 nAChR complexes with ligands were aligned and the root-mean-squared deviations (RMSDs) of these residues among the 17 ligand binding pockets were calculated using Pymol (https://www.pymol.org/). The receptor-ligand complexes were clustered according to their binding pocket residue RMSDs using the *hclust* tool (method: ward) in R (https://www.rdocumentation.org/packages/stats/versions/3.4.3/topics/hcl).

### Homology modeling of human α7 nAChR-LBD

The clustering results from the binding pocket analysis identified two distinct groups of binding pocket conformations among the 17 receptor-ligand complexes. One structure was selected from each of the two clusters (i.e., PDB IDs: 3SQ6 and 2XYT) as a template to construct the 3D structures of human α7 nAChR-LBD.

A series of homology modeling steps were performed using the Prime structure prediction wizard (http://www.schrodinger.com/Prime/) within Maestro (http://www.schrodinger.com/Maestro/) as follows. First, the primary sequences of the target protein and the template structure were compared. Sequence alignment was performed using ClustalW (http://www.ch.embnet.org/software/ClustalW.html) between the primary sequences of the two subunits (chain A and chain B) of human α7 nAChR (UniProt ID: P36544) and the template proteins 3SQ6 and 2XYT. The model was built based on the alignment between the target and template sequence with the ligands (EPJ from 3SQ6 and TC9 from 2XYT), through the knowledge-based and heteromultimer methods. The heteromultimer helps to build the model with more than one chains at a single run. There are four steps in the homology model construction. Initially the PRIME module copied the backbone atoms of the aligned region and the side chains of the conserved residues. Subsequently, the side chain was optimized and the non-template residues were minimized. Finally, the missing residues in the alignment file were inserted. The constructed human α7 nAChR-LBD structures based on 3SQ6 and 2XYT were minimized using the OPLS2005 force field. The minimized models were assessed using Ramachandran plots for the structural integrity and steric hindrance of the resulting.

### Structure refinement through MD simulations

The two 3D human α7 nAChR-LBD structures from homology modeling were subjected to MD simulations for structural optimization to remove any potential atomic clashes that might arise during construction. MD simulations were performed using Desmond from DE Shaw (https://www.deshawresearch.com/resources_desmond.html). OPLS2005 force field was used for both the proteins and ligands. In DESMOND, OPLS2005 has enough parameters to parametrize the ligands. The complex structures with OPLS force field and the DESMOND configuration file (parameter file) were used to run the molecular dynamics simulations. His residues in the modelled human α7 were single protonated. MD simulation system—consisting of an orthorhombic box with borders of 10Å from the receptor-ligand complex, containing simple point charge water molecules, and having a Na^+^ and Cl^−^ salt concentration of 0.15M—was constructed using System Builder. Prior to production simulations, both receptor-ligand systems were subjected to a relaxation stage using the default setting as follows: two steps of energy minimization of the protein–ligand complexes with and without restraint on the solute; 12 picosecond (ps) of constant number, volume, and temperature ensemble simulation carried out at 10 kelvin (K) with a Berendsen barostat and a fast temperature relaxation constant, velocity resampling set at every 1 ps, and restraints applied on all heavy atoms of the protein-ligand complex; 12 ps of constant number, pressure, and temperature (NPT) ensemble simulation carried out at 10 K, 1 atmospheric pressure (atm) using a Berendsen thermostat and barostat with a fast temperature relaxation constant to heat the system from 10 K to 300 K, and a slow pressure relaxation constant, velocity resampling set at every 1 ps, and restraints applied on all heavy atoms of the protein–ligand complexes; 12 ps of NPT simulation carried out at 300 K, 1 atm using the same thermostat, barostat, temperature relaxation constants, pressure relaxation constants, velocity resampling steps, and restraints; and finally, 24 ps of NPT simulation at 300 K, 1 atm using a fast temperature, and normal pressure relaxation constants. After this, production MD simulations were performed under NPT condition, 300 K, and pressure of 1 atm for 100 nanoseconds (ns) with energy being recorded at every 5 ps and trajectory at every 20 ps. The structural integrity of the final receptor structures obtained at the end of the MD simulations was checked using the Ramachandran plot.

### Preparation of ligands for docking

Two sets of ligands—training and test sets—were used in this study. The training set consisted of ligands extracted from the 17 receptor-ligand complexes obtained from the PDB. The ligands were imported to Maestro and checked for correct bond order and type before hydrogen atoms were added. The ligands in the test set were downloaded from PubChem (https://pubchem.ncbi.nlm.nih.gov/) in 3D SDF format. All ligands were protonated at pH 7.

### Preparation of human α7 nAChR-LBD structures for docking

A docking grid was generated around the binding pocket for the two human α7 nAChR-LBD structures constructed from homology modeling and refined by MD simulations. For the 3SQ6-based human α7 nAChR-LBD, the centroid of the ligand (EPJ) was set as the center of the grid and the size of the grid box was set to be suitable for ligands with a length of 10 Å. For the 2XYT-based human α7 nAChR-LBD, the centroid of the ligand (TC9) was set as the center of the grid, while the size of the grid box was set to be suitable for ligands with a length of 17 Å. Compared the residues in the active site between the two human α7 homology modeled protein to identify the conformation of the residues due to the ligand binding.

### Competitive docking model (CDM)

The ligands in the training set were docked to the grid boxes of the two 3D human α7 nAChR-LBD structures using Glide [72–74] in Maestro (http://www.schrodinger.com/Glide). The extra precision docking method was used while the rest of the docking parameters were maintained as default: van der Waals radii scaling factor of 0.80, partial charge cutoff of 0.15, flexible ligand sampling with regard to nitrogen inversions and ring conformations, and Epik state penalties applied to the docking scores. The number of ligand poses to include for post-docking minimization was set at five, and only one pose per ligand was written out. Three possible scenarios were anticipated from the docking of a ligand to the two human α7 nAChR-LBD structures: (1) the ligand was successfully docked to only one of the two structures: the 3SQ6-based or the 2XYT-based human α7 nAChR-LBD structure; (2) the ligand was successfully docked to both structures; and (3) the ligand could not be docked to either structure. In scenario (1), the ligand would be considered to bind human α7 nAChR in the conformation similar to the 3SQ6-based or 2XYT-based human α7 nAChR-LBD structure; therefore, the associated docking score would be used for the CDM development. In scenario (2), the ligand would be considered to bind the human α7 nAChR-LBD structure whose docking score was the lower of the two (more favorable), as obtained from the ligand docking to the 3SQ6-based and 2XYT-based human α7 nAChR-LBD structures, and the lower docking score would be used in the CDM. In scenario (3), the ligand would be considered as a non-binder of human α7 nAChR. Based on this rule, each ligand from the training set would be associated with a favored docked complex and docking score (or none if it was a non-binder). The predictive CDM was developed using these docking scores according to equation (2), below:

$$\text{CDM}=1-\frac{\text{score}-\text{score lowest}}{\text{score highest}-\text{score lowest}}$$ (2)

‘Score’ indicates the winning docking score (the lower docking score between the two human α7 nAChR for the same chemical), while ‘score highest’ and ‘score lowest’ are the highest and lowest winning scores among the 17 scores. When the CDM value calculated from docking is less than zero, the chemical is predicted to be a non-binder of human α7 nAChR; the chemical is predicted to be a binder if its CDM value is greater than or equal to zero.

### Elucidation of key binding interactions through MD simulations

In order to define the potential key binding interactions between human α7 nAChR-LBD and its binding ligands, the 17 complexes obtained from the CMD were subjected to MD simulations. The MD simulations were performed for each complex in a manner similar to the human α7 nAChR-LBD structure-refinement step. First, a relaxation stage identical to what was described in 3D structure optimization was performed; next, a production of 20 ns of MD simulations was performed. All simulation parameters remained unchanged. To assess simulation stability, the RMSD of the 20 ns simulations was plotted against time. The key binding interactions (i.e., hydrophobic contact, pi-pi interactions, pi-cation and hydrogen bonds, root-mean-squared fluctuations [RMSFs] of the residues) were investigated using the frames obtained from the last ns of the MD simulations. These analyses were performed using the simulation interactions diagram tool in Maestro (http://www.schrodinger.com/Maestro/).

### Binding activity predictions of the compounds for experimental validation

After development of the CDM, the compounds in the test set were docked to the two 3D human α7 nAChR-LBD structures, and their respective favored docking scores were used to make predictions, based on equation (1), of the binding activity of the respective compounds.

### Experimental validation

The CDM performance in the prediction of ligand binding activity was assessed by comparing the prediction results with the findings obtained from experimental testing.

A Center for Tobacco Products (CTP) sent 12 compounds for blind testing of the CDM. The 12 compounds were assayed for their activity to the human α7 nAChRs subtype in a CTP-contracted project in which 786 compounds were profiled in five human nicotinic subtypes including the α7 [75]. The results were sent to the CTP scientist for assessing CDM performance.

## SUPPLEMENTARY MATERIALS FIGURES



## Abbreviations

AChBP: Acetylcholine binding protein
Atm: Atmospheric pressure
CDM: Competitive docking model
HPHCs: Harmful and potentially harmful constituents
LBD: Ligand-binding domain
MD: Molecular dynamics
nAChRs: Nicotinic acetylcholine receptors
ns: Nano second
ps: Pico second
RMSDs: Root mean squared deviation
RMSFs: Root mean squared fluctuations
