# Supplementary material for: Competitive docking model for prediction of the human nicotinic acetylcholine receptor α7 binding of tobacco constituents
Source: Oncotarget. 2018 Feb 8;9(24):16899–916. doi: 10.18632/oncotarget.24458 (PMC5908294; doi:10.18632/oncotarget.24458)
Supplement: Supplementary file 1 [file oncotarget-09-16899-s001.pdf]

Competitive docking model for prediction of the human nicotinic acetylcholine receptor α7 binding of tobacco constituents

SUPPLEMENTARY MATERIALS

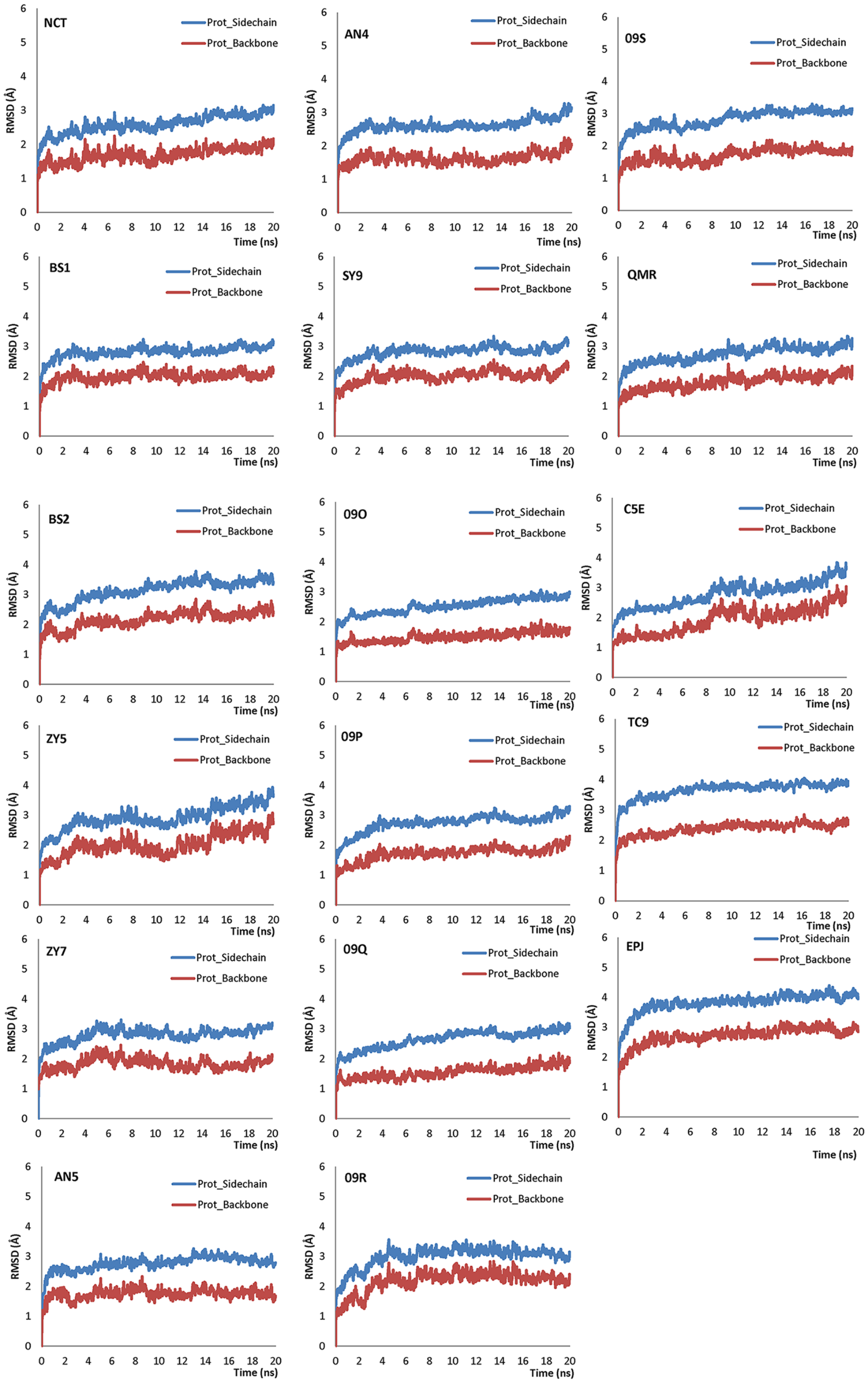

Supplementary Figure 1: RMSDs of the human α7 nAChR-LBD structure side chain (blue) and backbone (red) bound to the training set ligands.

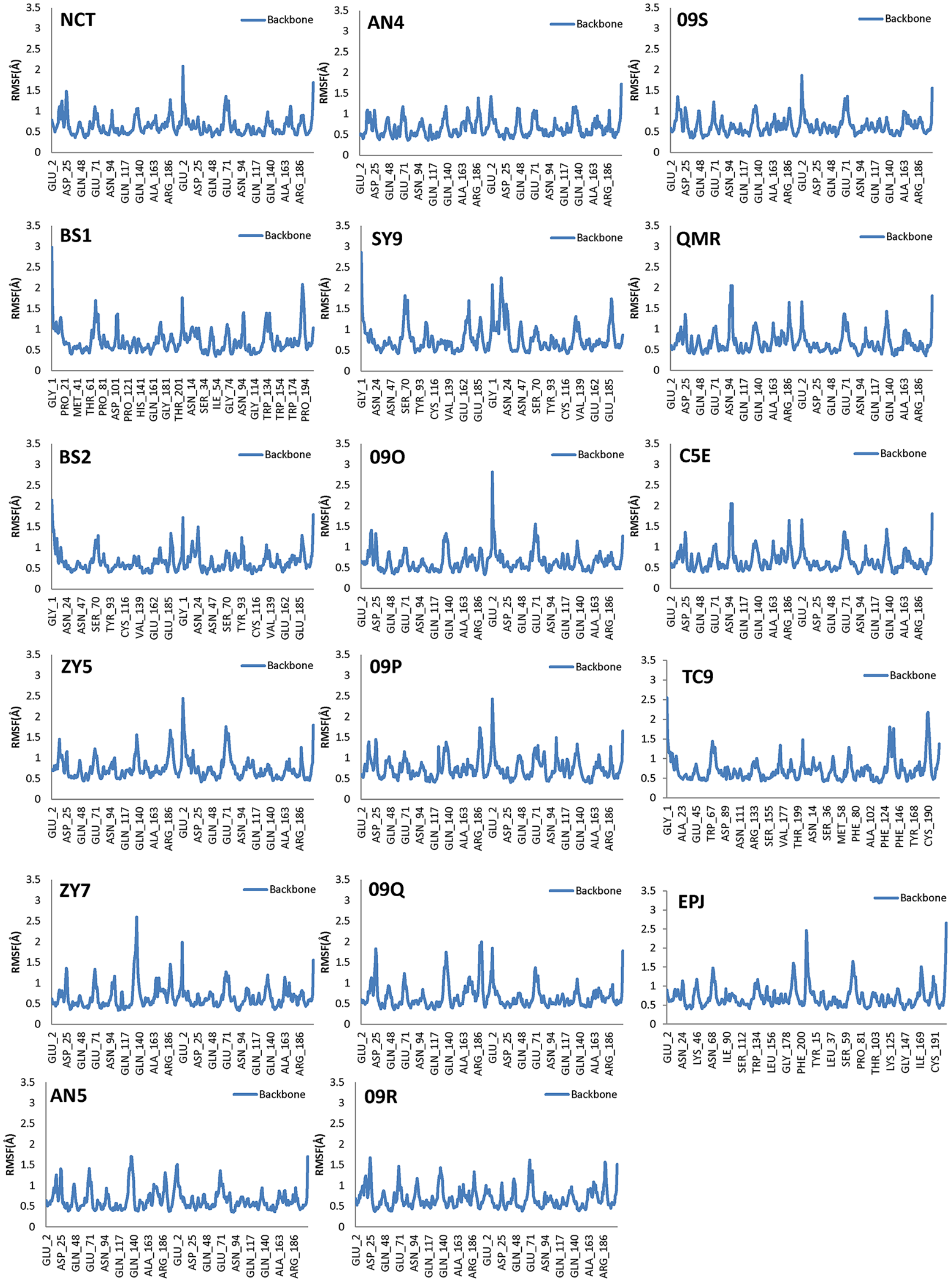

Supplementary Figure 2: RMSFs of the backbone of the human α7 nAChR-LBD structure bound to the training set ligands.

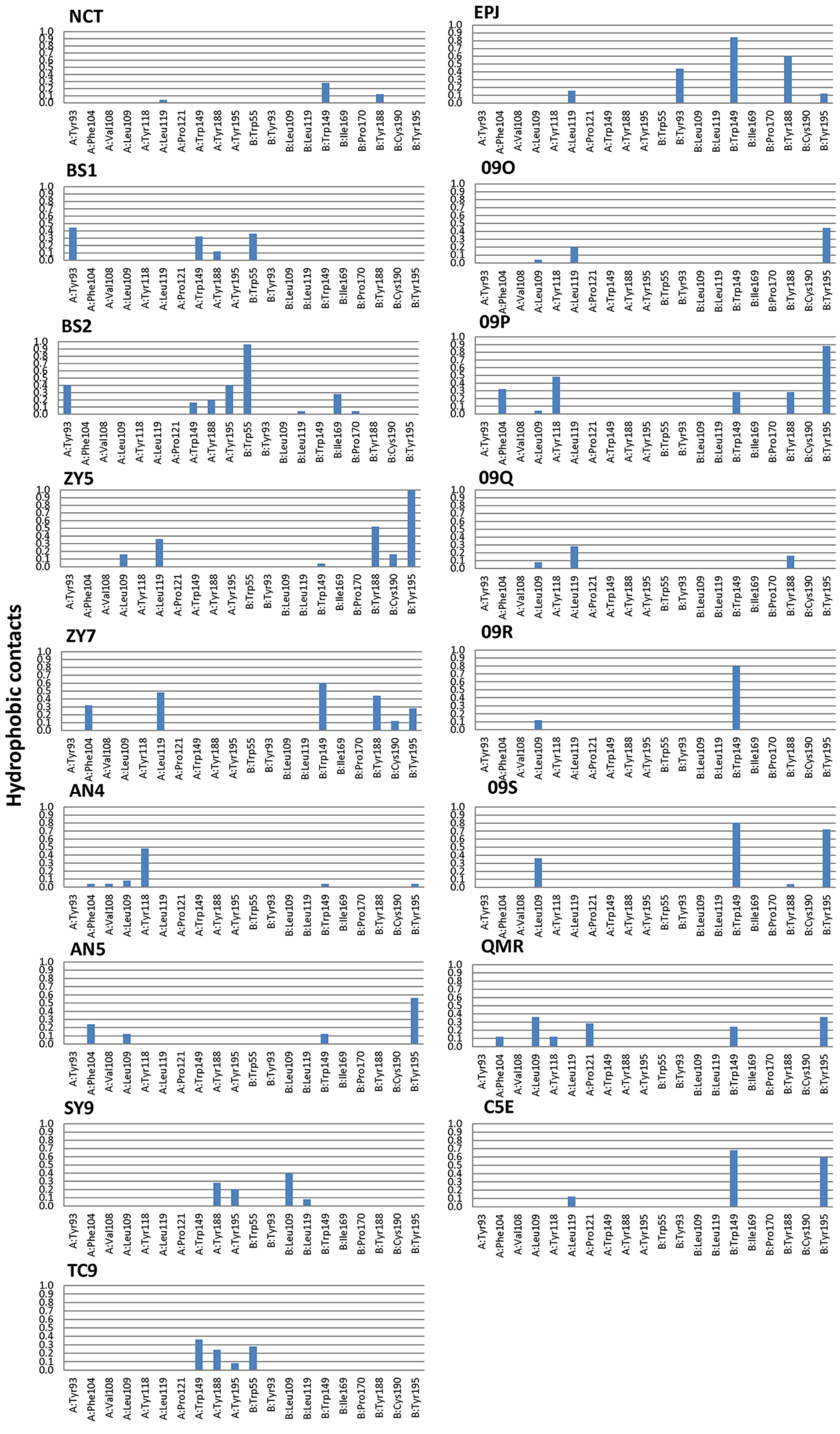

Supplementary Figure 3: Hydrophobic contacts established between the training set ligands and the human α7 nAChR-LBD structure, expressed as fractions, calculated from frames obtained from the final ns trajectory of the MD simulations.

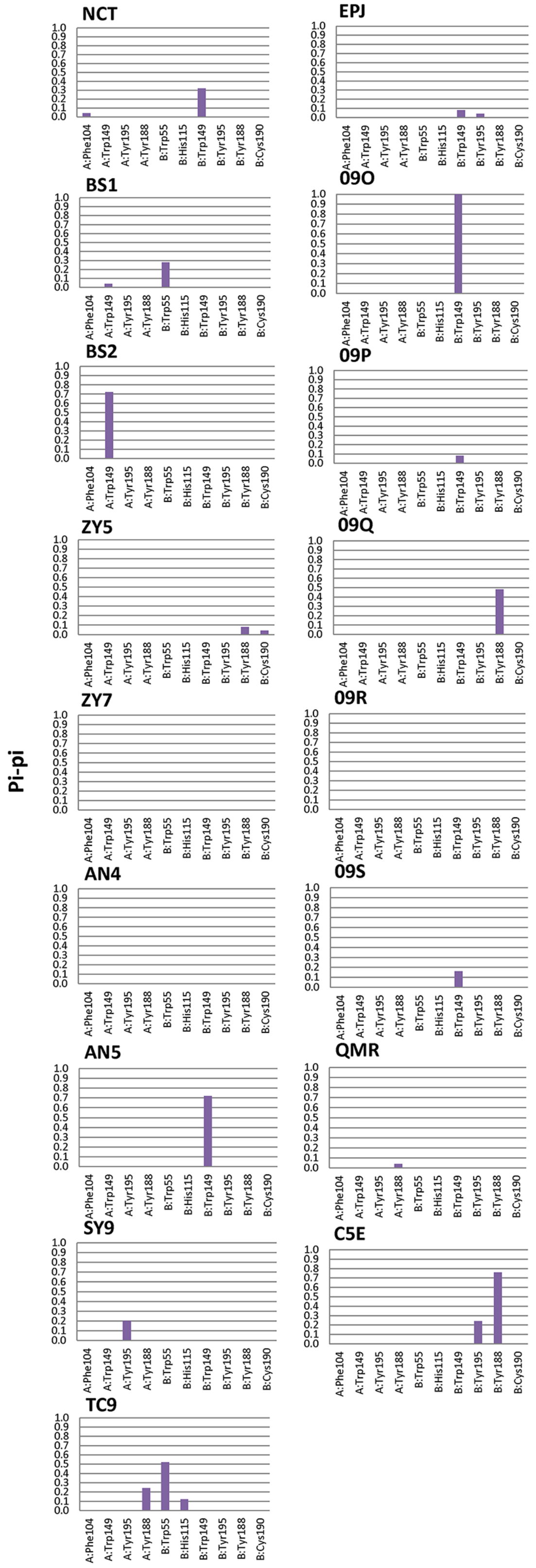

Supplementary Figure 4: Pi-pi interactions established between the training set ligands and the human α7 nAChR-LBD structure, expressed as fractions, calculated from frames obtained from the final ns trajectory of the MD simulations.

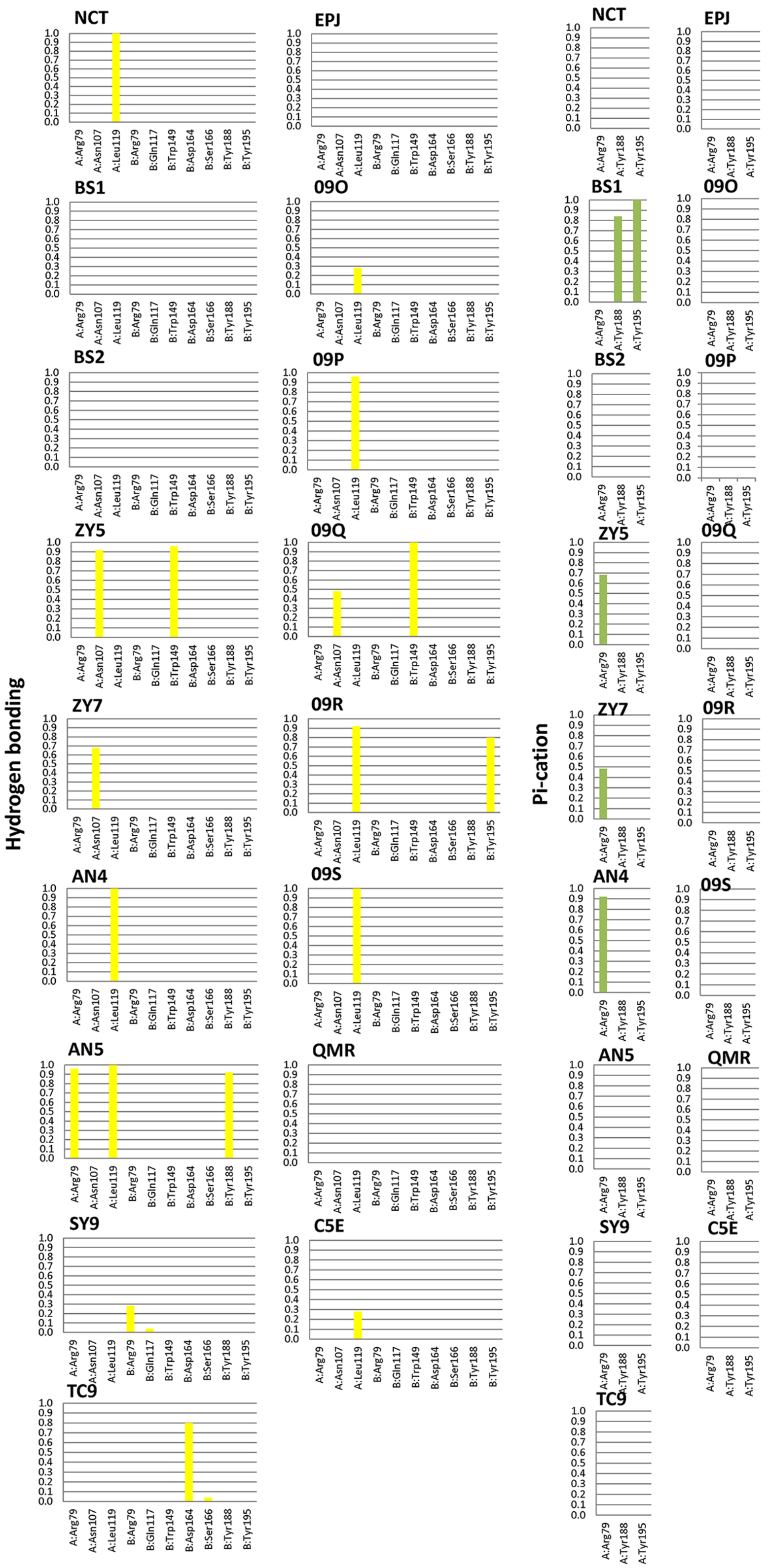

Supplementary Figure 5: Hydrogen bonding and pi-cation interactions established between the training set ligands and the human α7 nAChR-LBD structure, expressed as fractions, calculated from frames obtained from the final ns trajectory of the MD simulations.

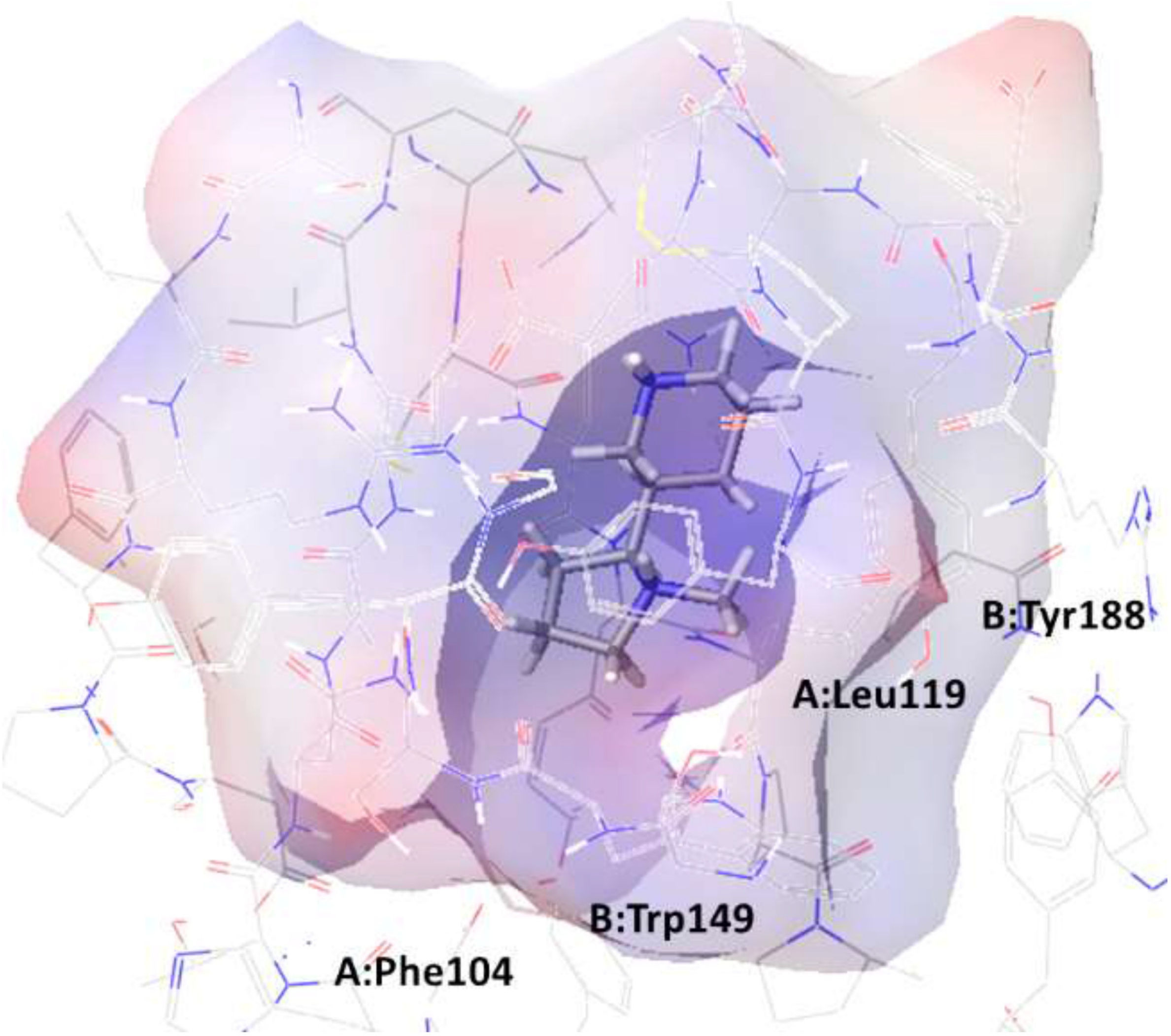

Supplementary Figure 6: Interactions established between NCT and the human α7 nAChR-LBD structure. The annotated residues indicate the key interacting residues and the electrostatic potential of the surface of the pocket are shown (red indicates electronegative surface while blue indicates electropositive surface).

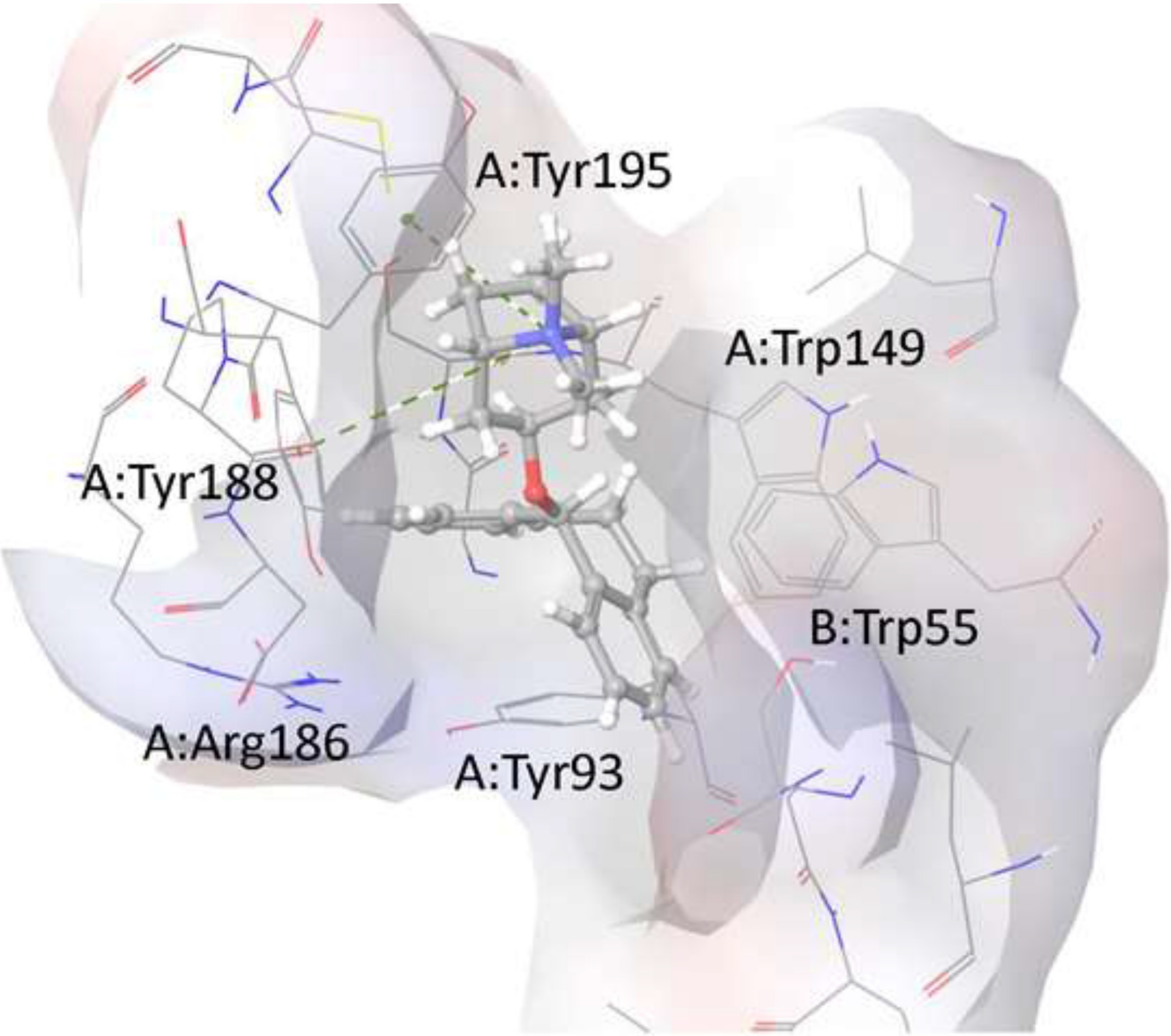

Supplementary Figure 7: Interactions established between BS1 and the human α7 nAChR-LBD structure. The annotated residues indicate the key interacting residues and the electrostatic potential of the surface of the pocket are shown (red indicates electronegative surface while blue indicates electropositive surface). The green dotted lines indicate pi-cation interactions.

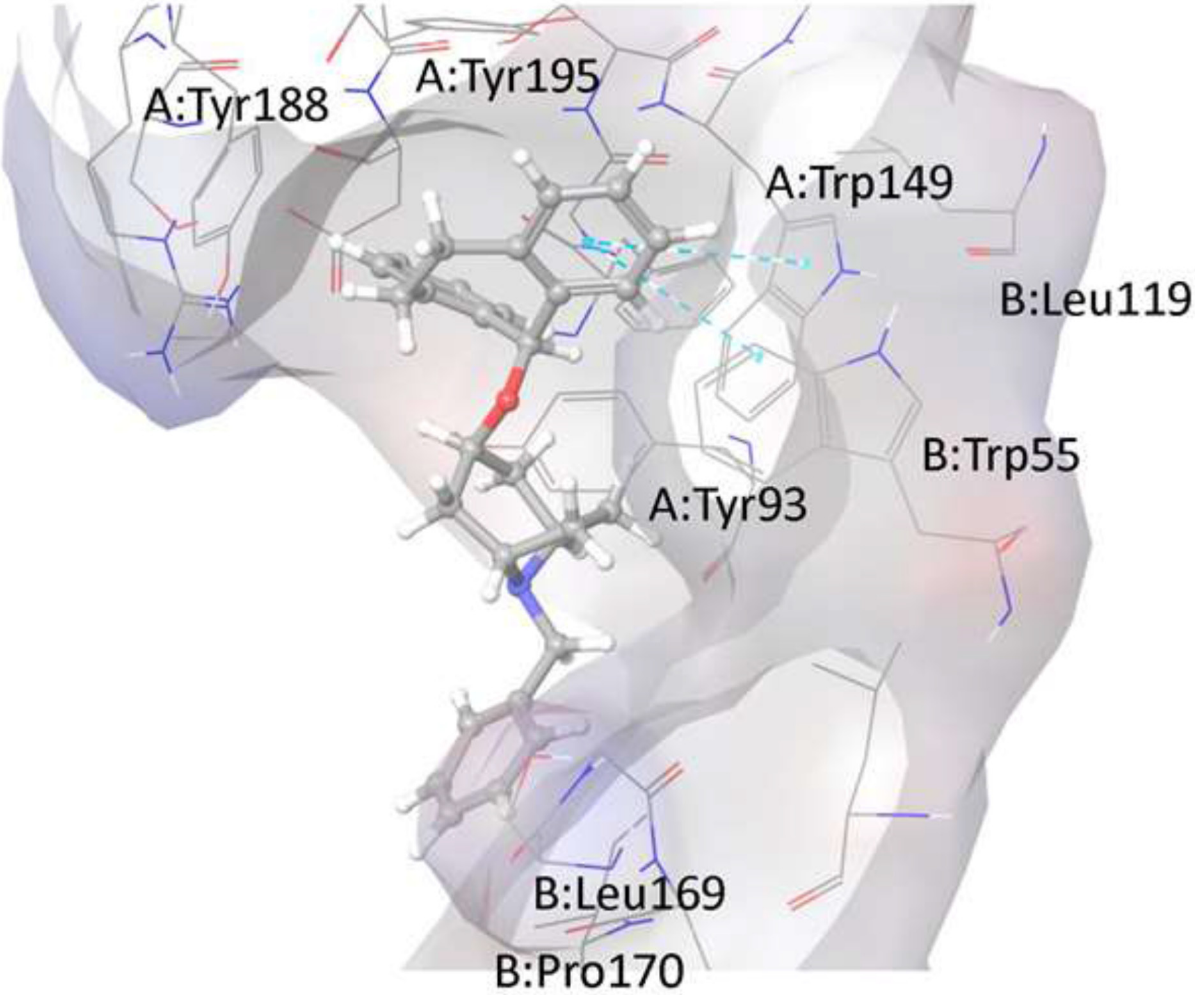

Supplementary Figure 8: Interactions established between BS2 and the human α7 nAChR-LBD structure. The annotated residues indicate the key interacting residues and the electrostatic potential of the surface of the pocket are shown (red indicates electronegative surface while blue indicates electropositive surface). The blue dotted lines indicate pi-pi interactions.

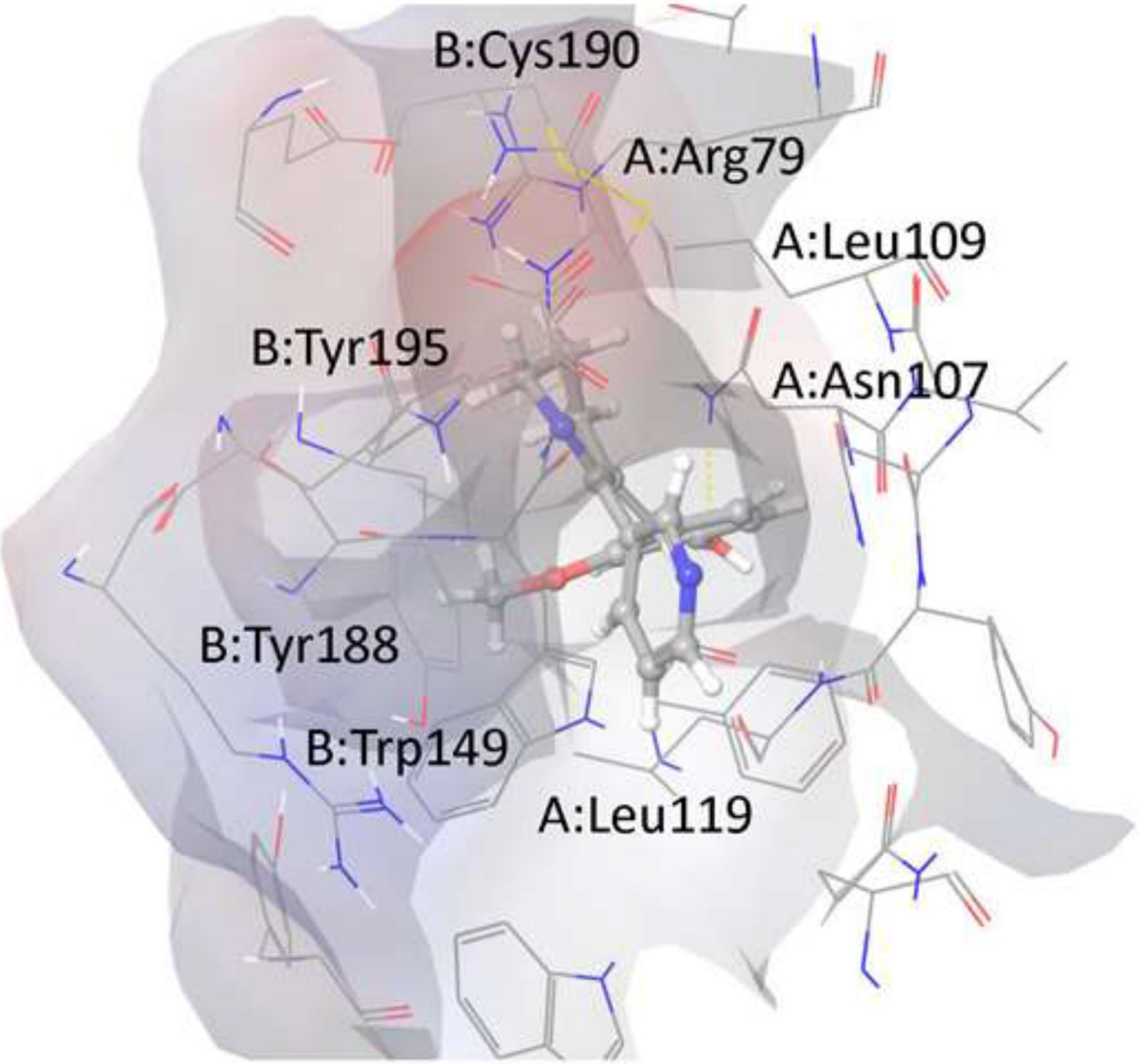

Supplementary Figure 9: Interactions established between ZY5 and the human α7 nAChR-LBD structure. The annotated residues indicate the key interacting residues and the electrostatic potential of the surface of the pocket are shown (red indicates electronegative surface while blue indicates electropositive surface).

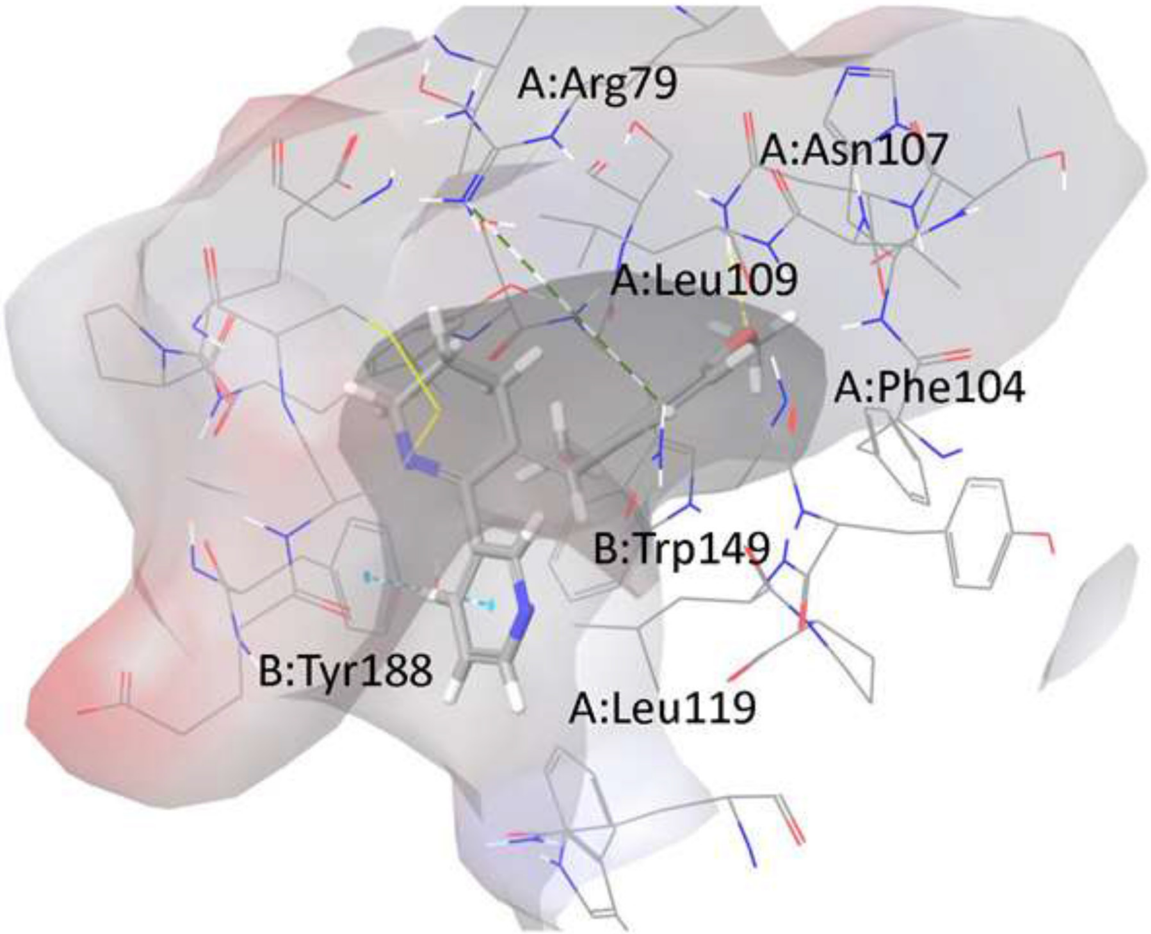

Supplementary Figure 10: Interactions established between ZY7 and the human α7 nAChR-LBD structure. The annotated residues indicate the key interacting residues and the electrostatic potential of the surface of the pocket are shown (red indicates electronegative surface while blue indicates electropositive surface). The green dotted line indicates pi-cation interactions while the yellow and blue dotted line indicates hydrogen bonding and pi-pi interactions respectively.

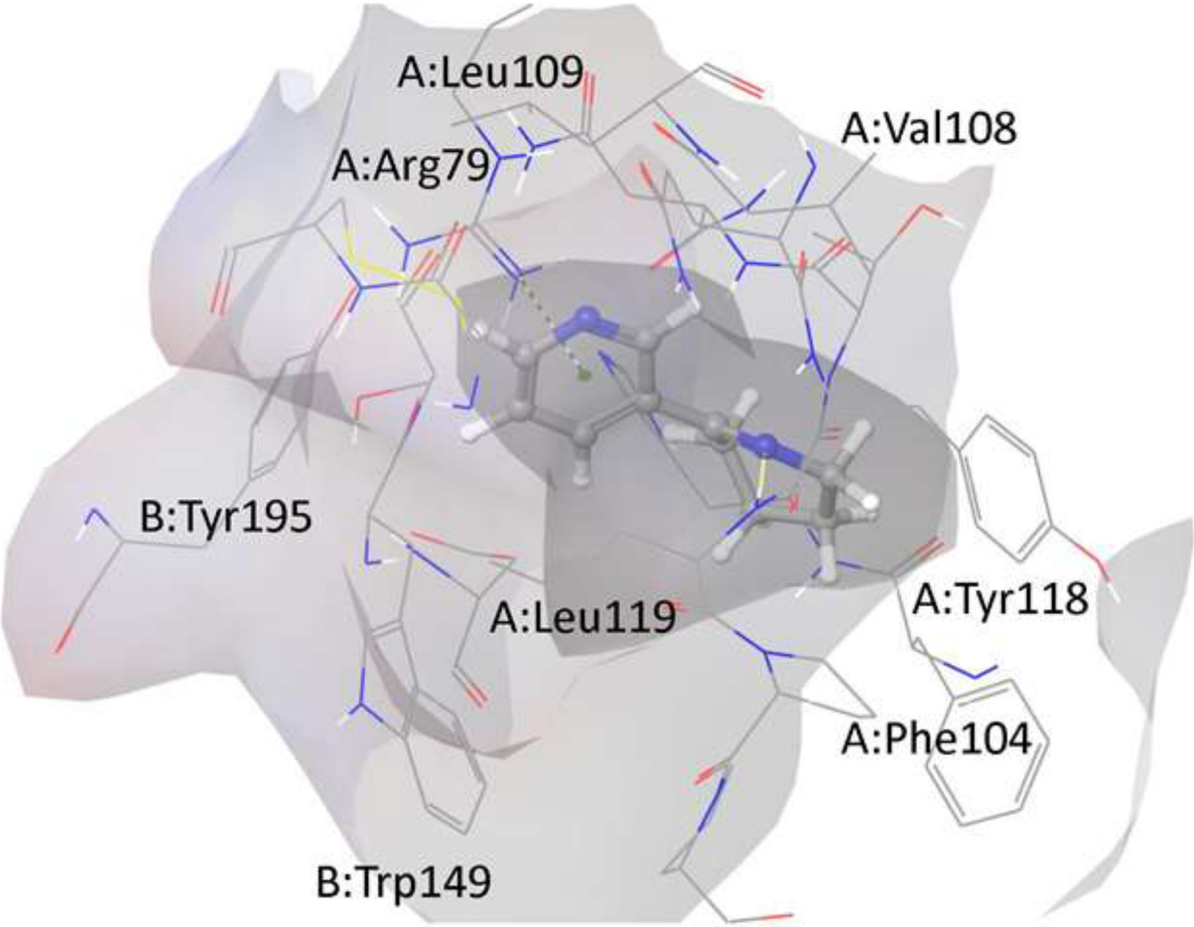

Supplementary Figure 11: Interactions established between AN4 and the human α7 nAChR-LBD structure. The annotated residues indicate the key interacting residues and the electrostatic potential of the surface of the pocket are shown (red indicates electronegative surface while blue indicates electropositive surface). The green dotted line indicates pi-cation interactions while the yellow dotted line indicates hydrogen bonding.

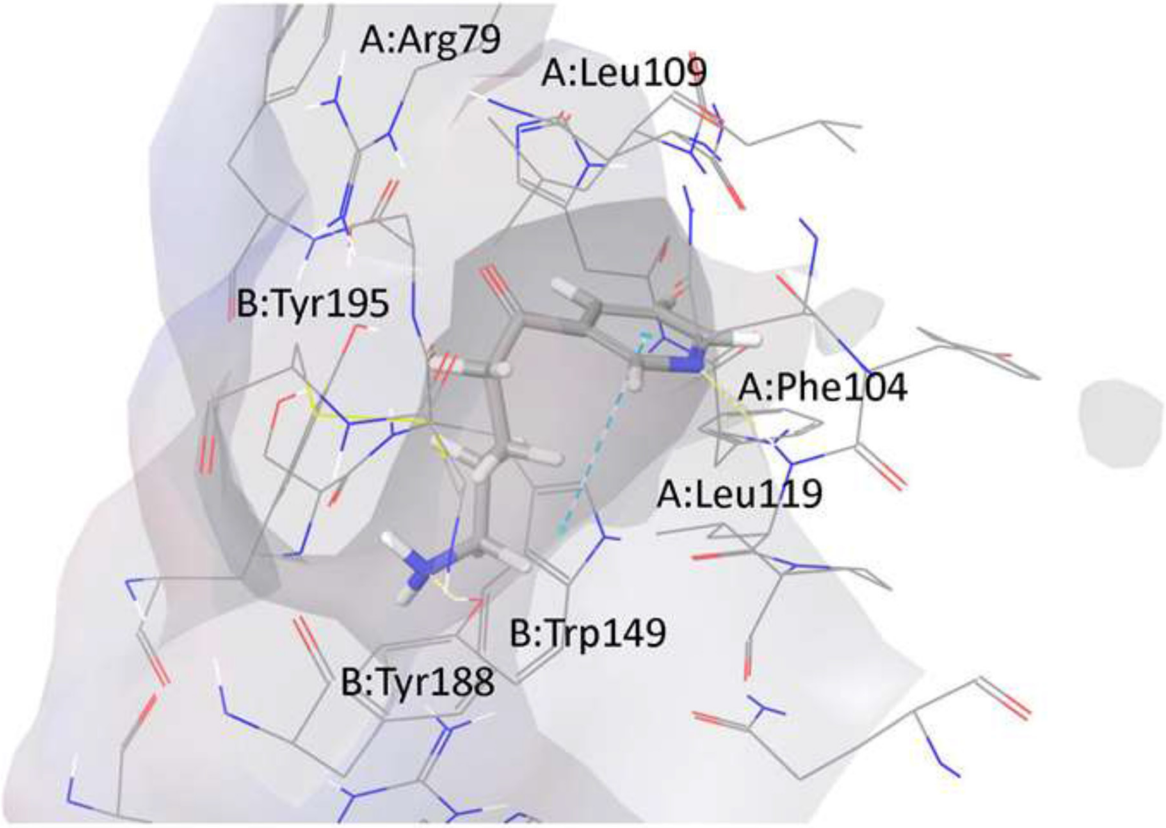

Supplementary Figure 12: Interactions established between AN5 and the human α7 nAChR-LBD structure. The annotated residues indicate the key interacting residues and the electrostatic potential of the surface of the pocket are shown (red indicates electronegative surface while blue indicates electropositive surface). The blue dotted line indicates pi-pi interactions.

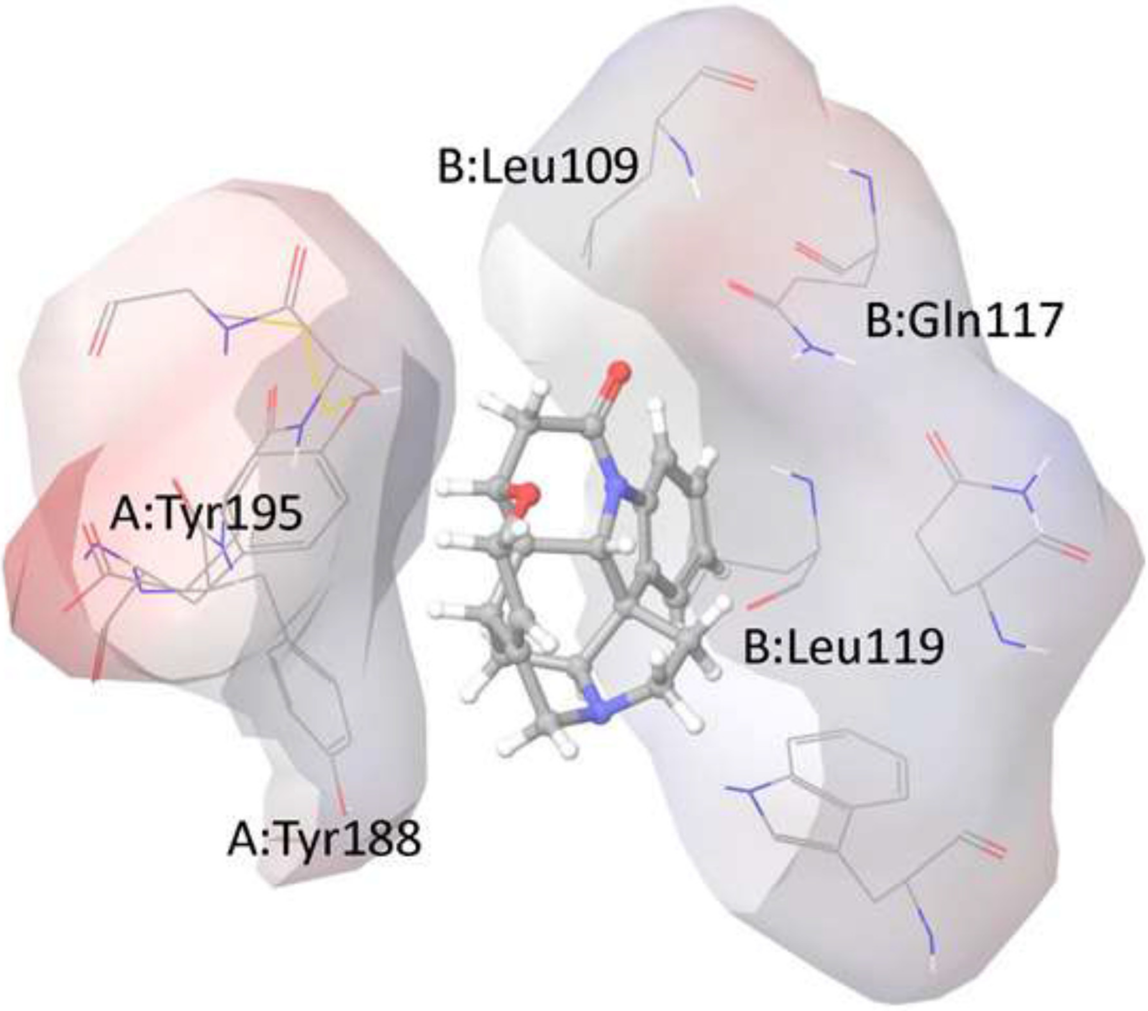

Supplementary Figure 13: Interactions established between SY9 and the human α7 nAChR-LBD structure. The annotated residues indicate the key interacting residues and the electrostatic potential of the surface of the pocket are shown (red indicates electronegative surface while blue indicates electropositive surface).

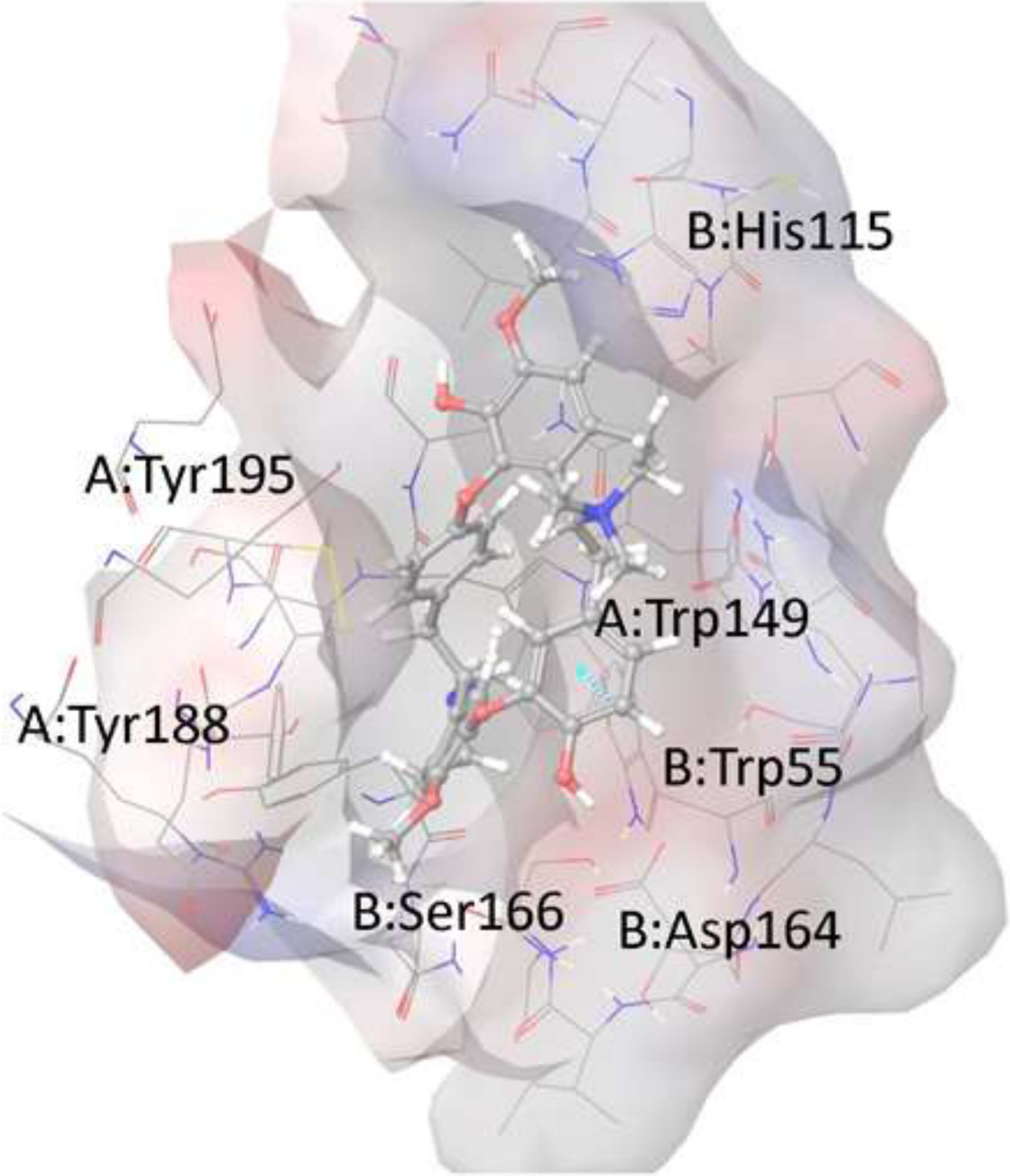

Supplementary Figure 14: Interactions established between TC9 and the human α7 nAChR-LBD structure. The annotated residues indicate the key interacting residues and the electrostatic potential of the surface of the pocket are shown (red indicates electronegative surface while blue indicates electropositive surface). The blue dotted line indicates pi-pi interactions.

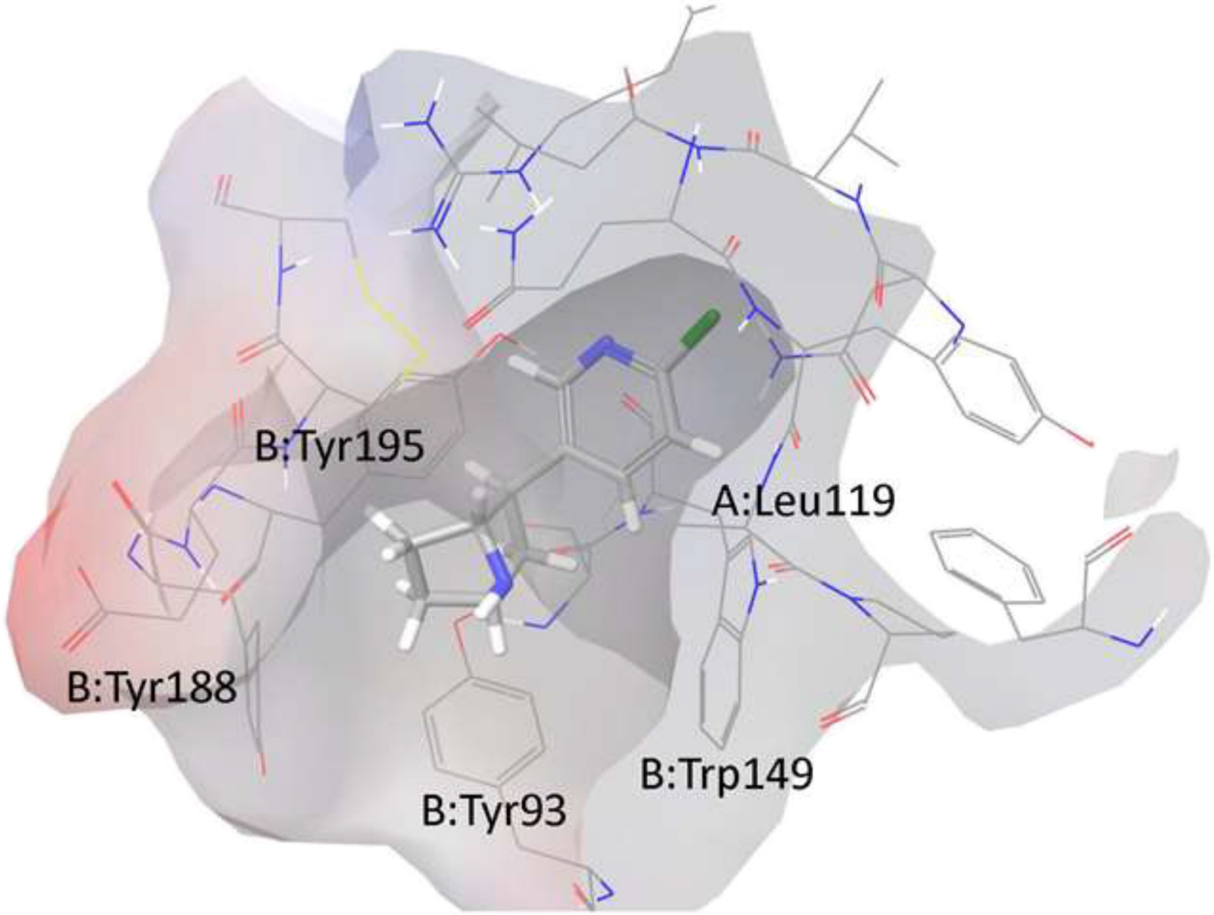

Supplementary Figure 15: Interactions established between EPJ and the human α7 nAChR-LBD structure. The annotated residues indicate the key interacting residues and the electrostatic potential of the surface of the pocket are shown (red indicates electronegative surface while blue indicates electropositive surface).

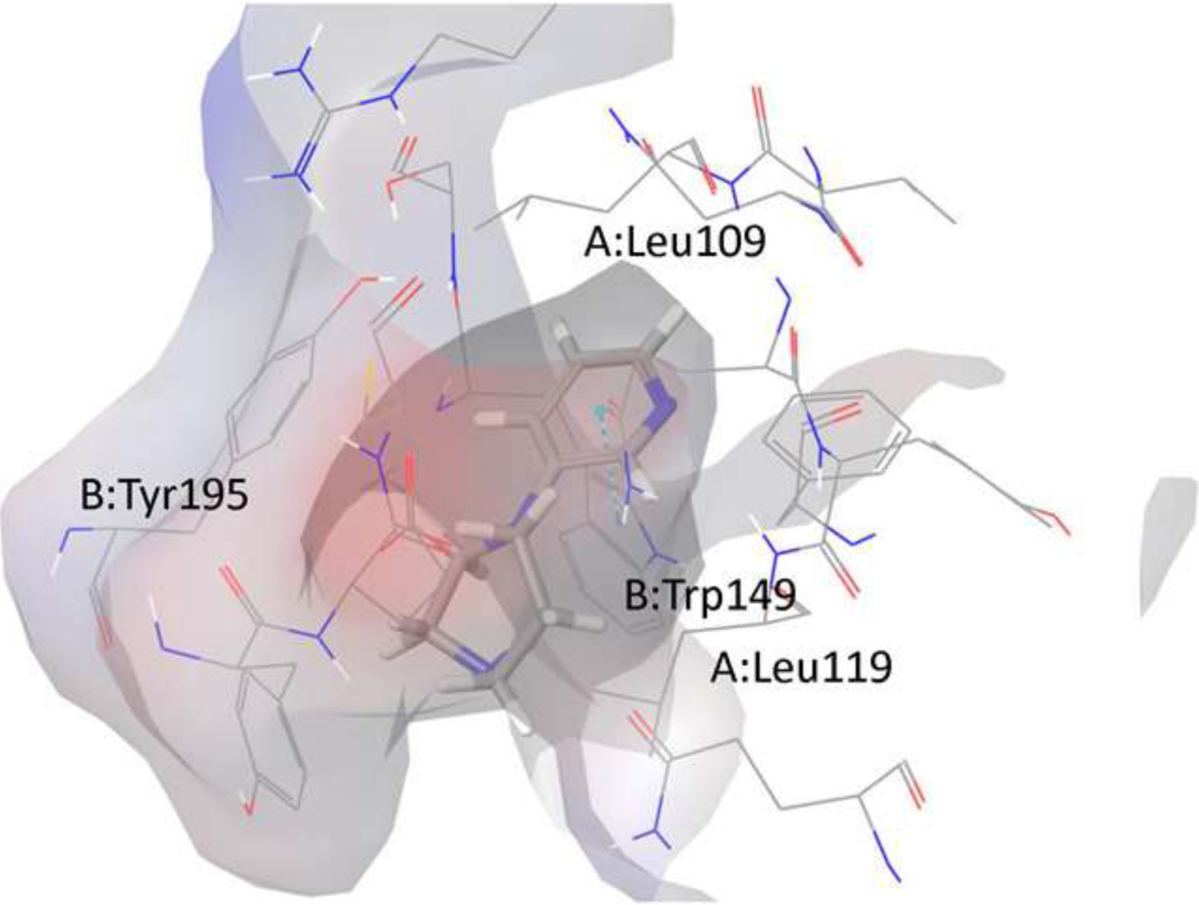

Supplementary Figure 16: Interactions established between 09O and the human α7 nAChR-LBD structure. The annotated residues indicate the key interacting residues and the electrostatic potential of the surface of the pocket are shown (red indicates electronegative surface while blue indicates electropositive surface). The blue dotted line indicates pi-pi interactions.

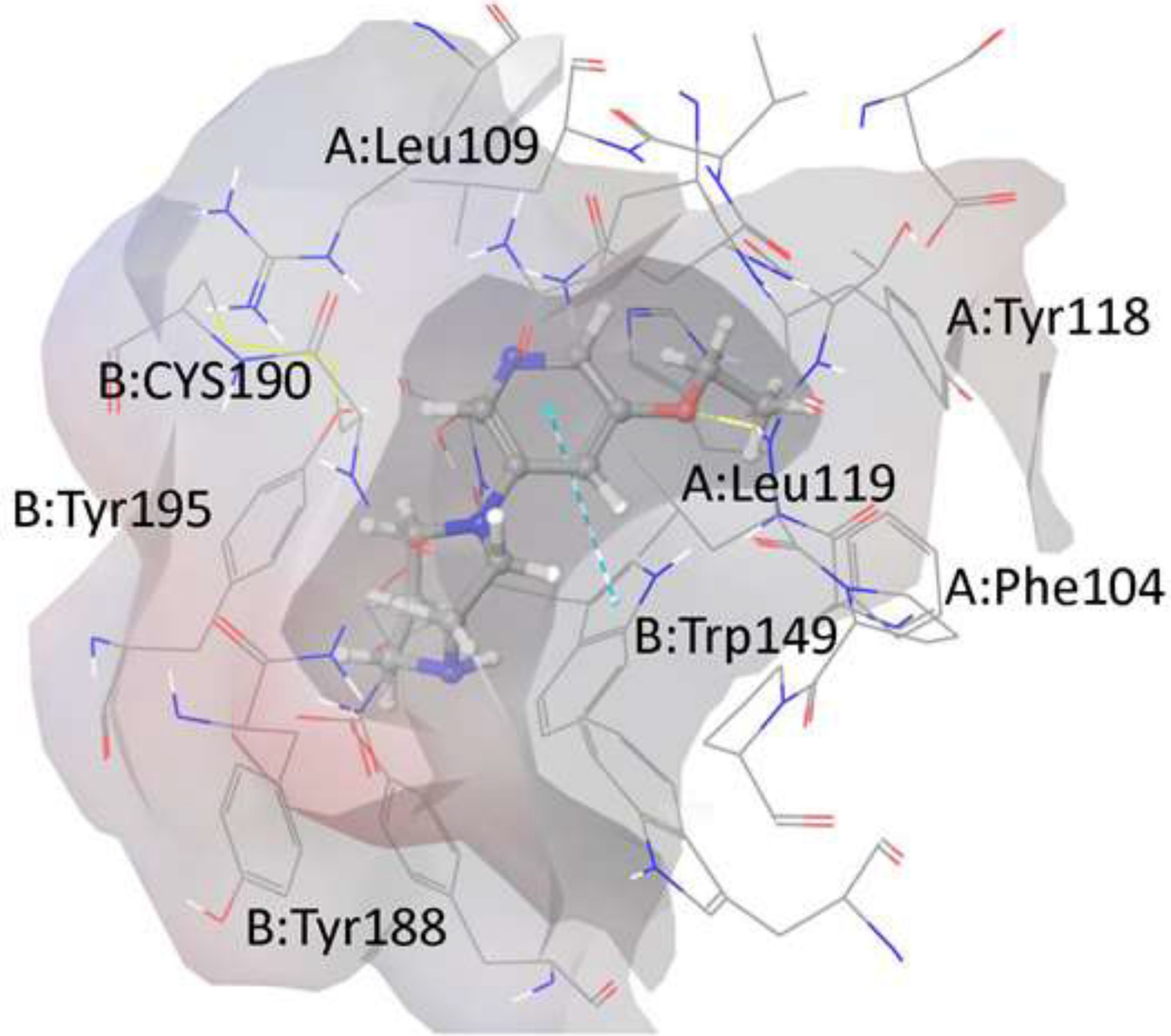

Supplementary Figure 17: Interactions established between 09S and the human α7 nAChR-LBD structure. The annotated residues indicate the key interacting residues and the electrostatic potential of the surface of the pocket are shown (red indicates electronegative surface while blue indicates electropositive surface). The blue dotted line indicates pi-pi interactions while the yellow dotted line indicates hydrogen bonding.

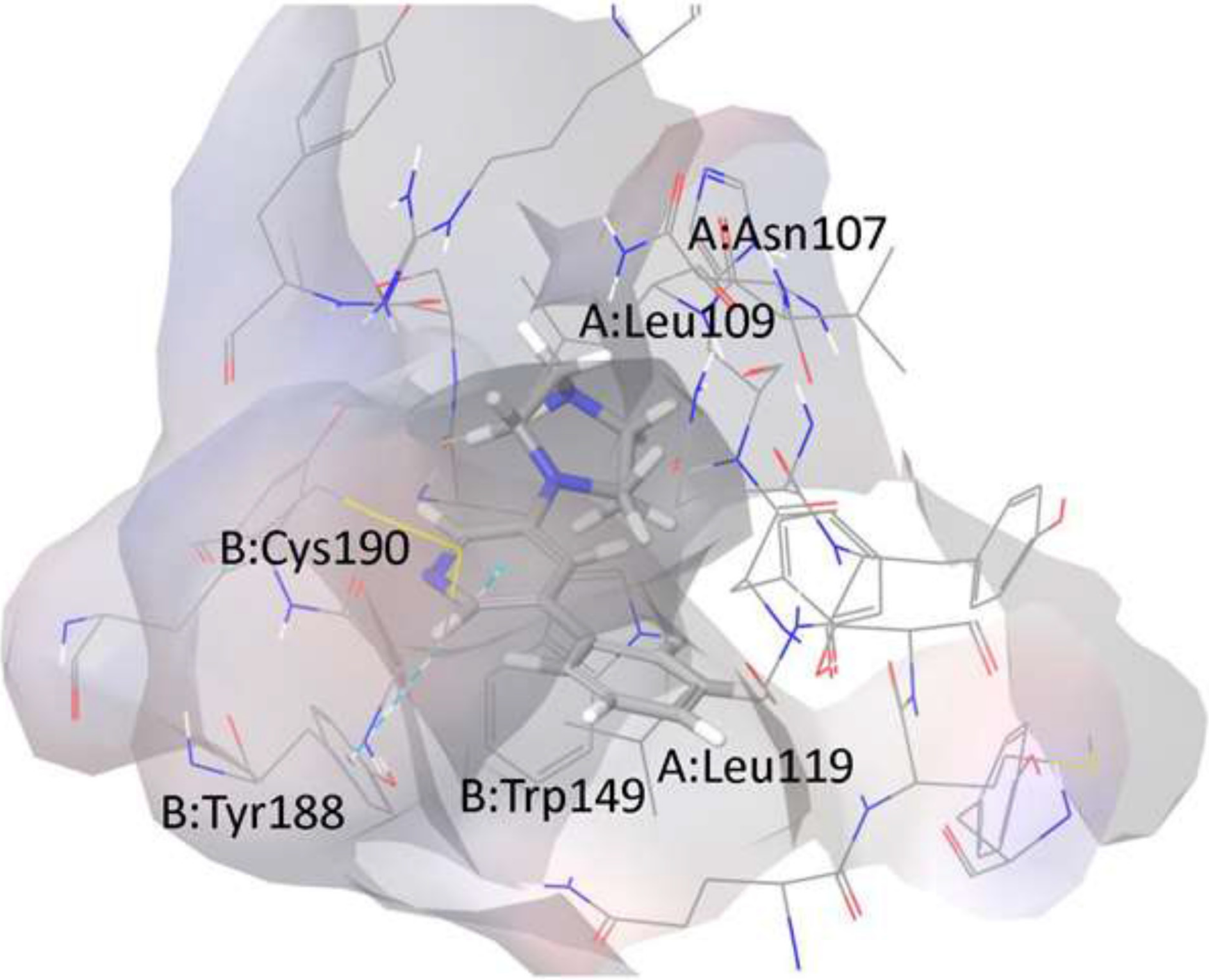

Supplementary Figure 18: Interactions established between 09Q and the human α7 nAChR-LBD structure. The annotated residues indicate the key interacting residues and the electrostatic potential of the surface of the pocket are shown (red indicates electronegative surface while blue indicates electropositive surface). The blue dotted line indicates pi-pi interactions.

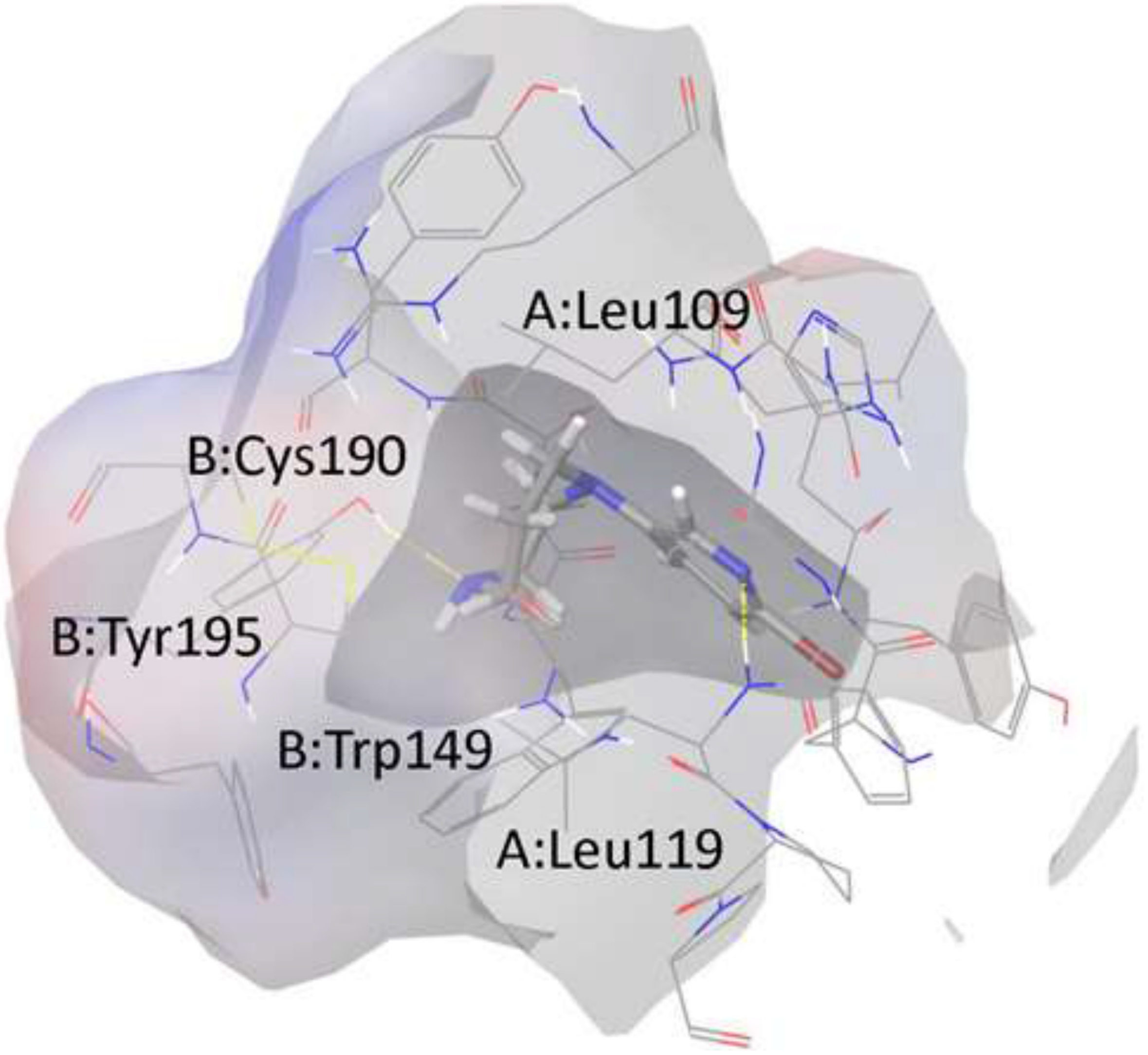

Supplementary Figure 19: Interactions established between 09R and the human α7 nAChR-LBD structure. The annotated residues indicate the key interacting residues and the electrostatic potential of the surface of the pocket are shown (red indicates electronegative surface while blue indicates electropositive surface). The yellow dotted lines indicate hydrogen bonding.

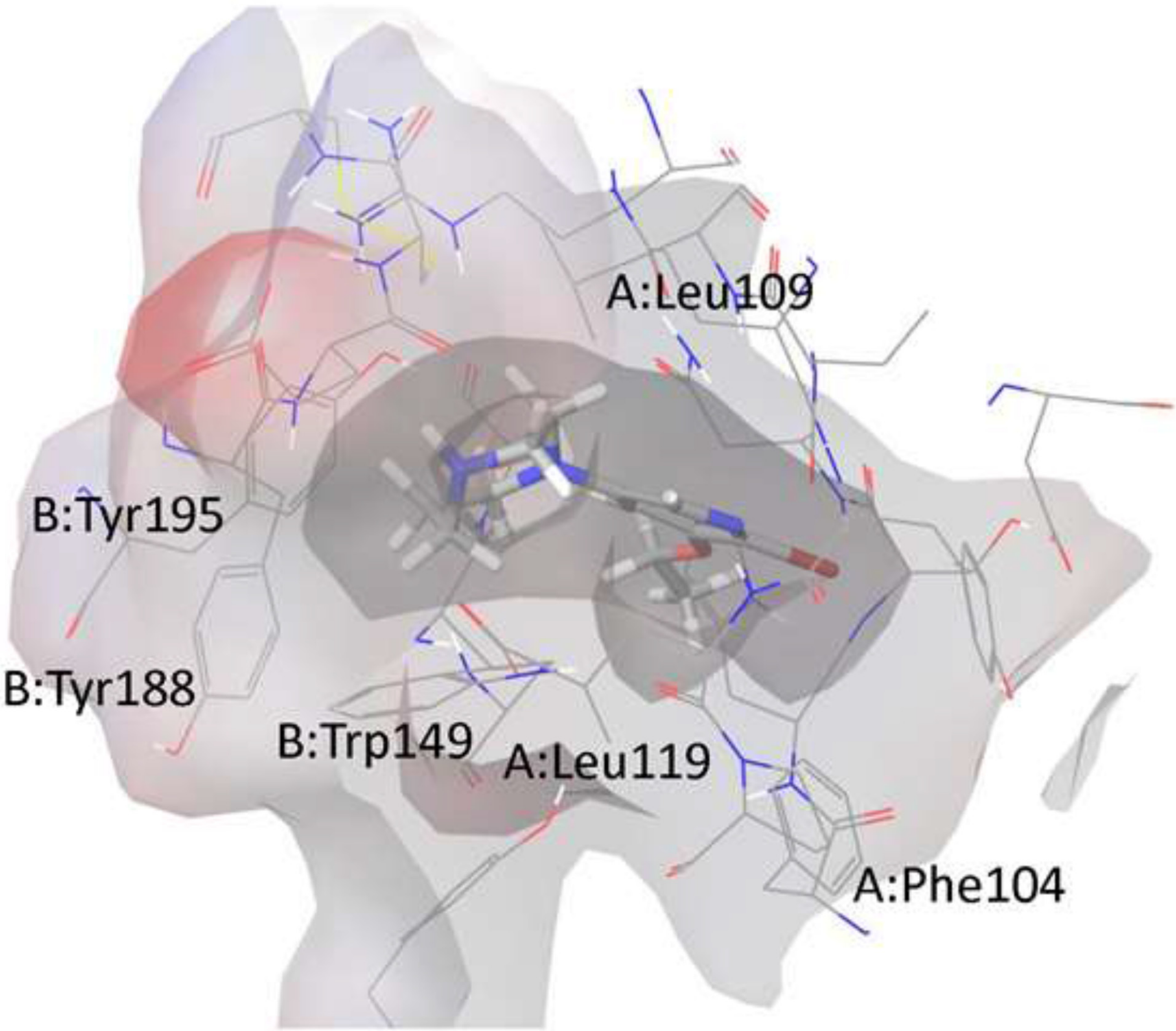

Supplementary Figure 20: Interactions established between 09P and the human α7 nAChR-LBD structure. The annotated residues indicate the key interacting residues and the electrostatic potential of the surface of the pocket are shown (red indicates electronegative surface while blue indicates electropositive surface).

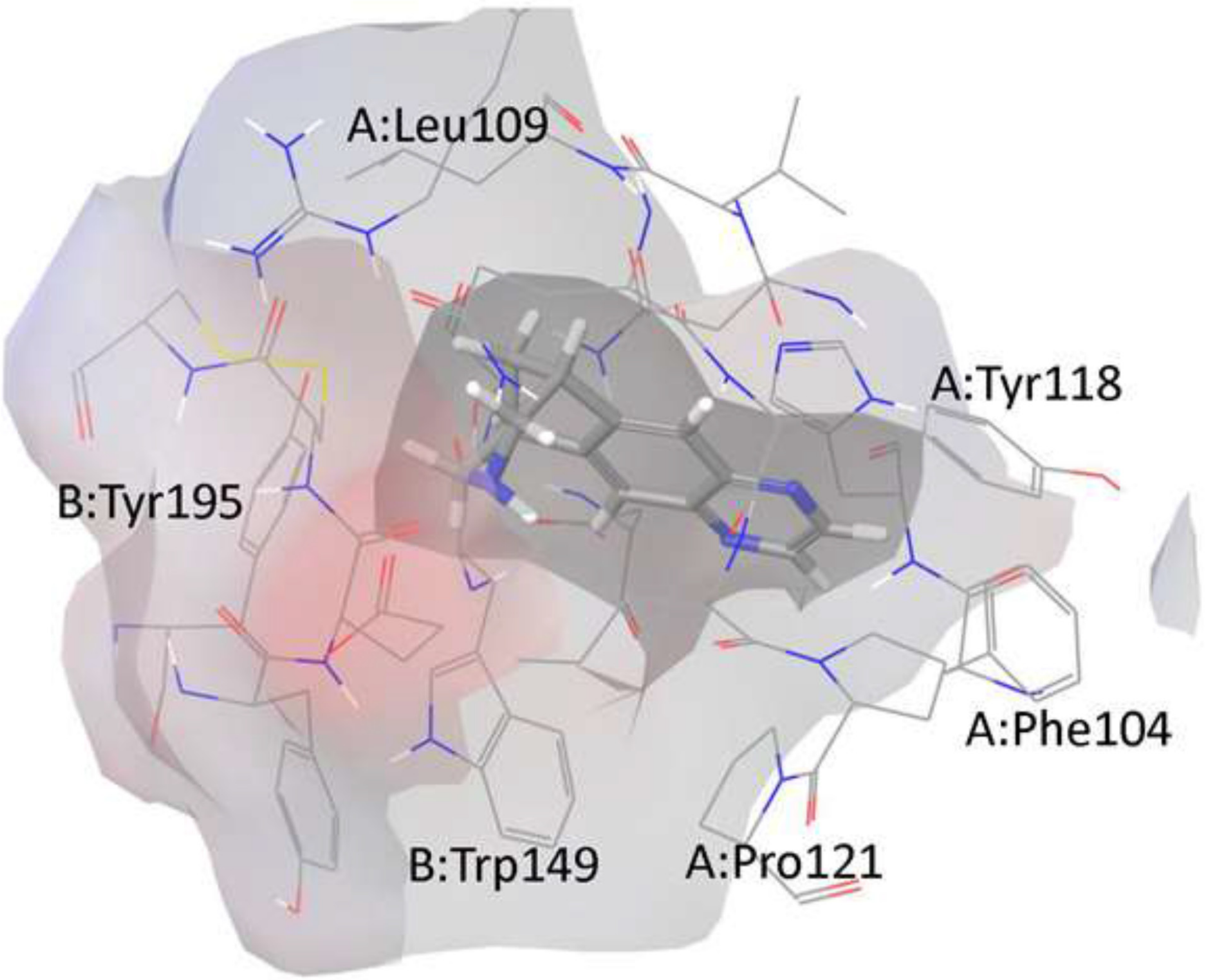

Supplementary Figure 21:Interactions established between QMR and the human α7 nAChR-LBD structure. The annotated residues indicate the key interacting residues and the electrostatic potential of the surface of the pocket are shown (red indicates electronegative surface while blue indicates electropositive surface).

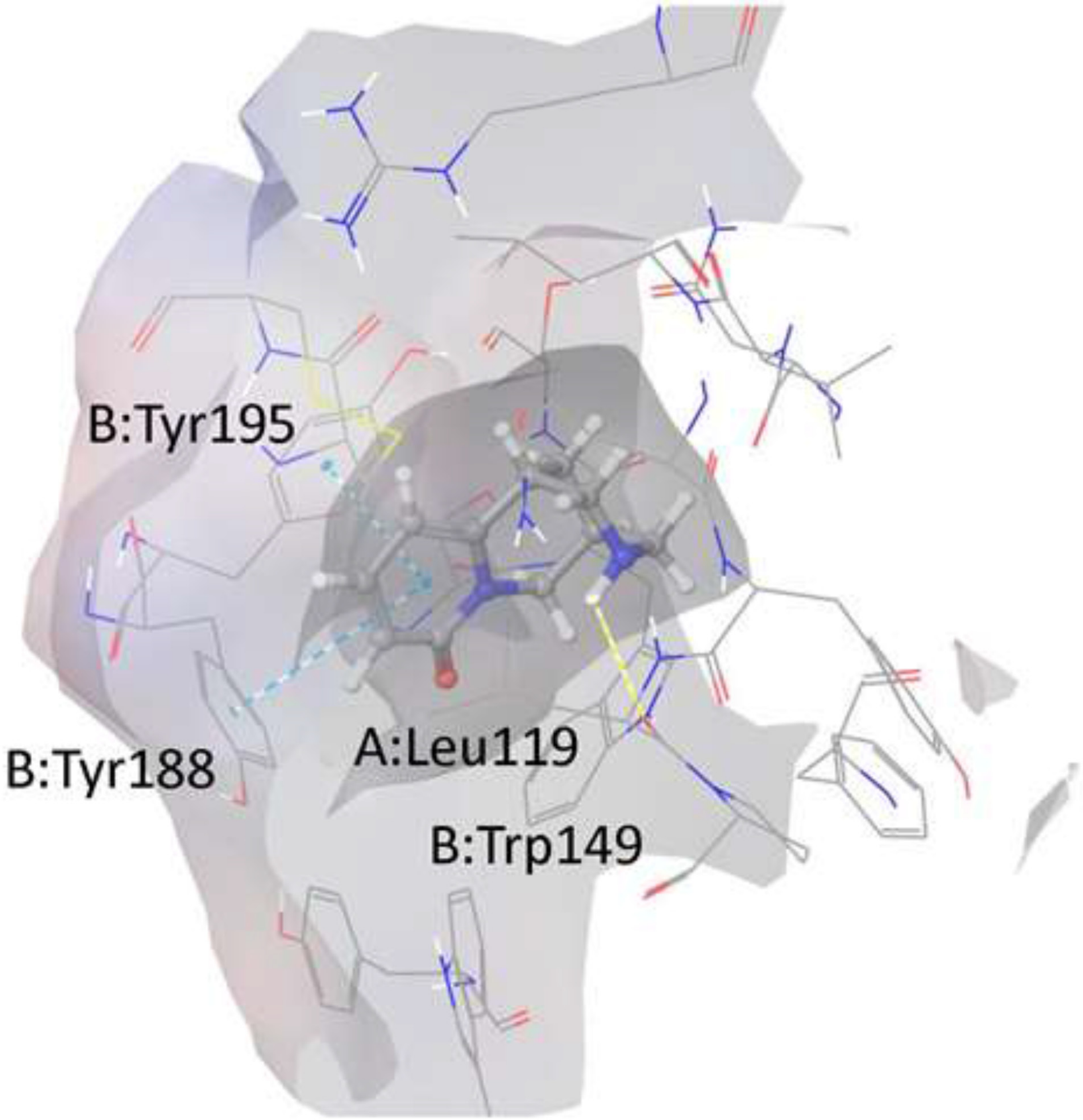

Supplementary Figure 22: Interactions established between C5E and the human α7 nAChR-LBD structure. The annotated residues indicate the key interacting residues and the electrostatic potential of the surface of the pocket are shown (red indicates electronegative surface while blue indicates electropositive surface). The blue dotted lines indicate pi-pi interactions while the yellow dotted line indicates hydrogen bonding.
